# Adaptive prospective optical gating enables day-long 3D time-lapse imaging of the beating embryonic zebrafish heart

**DOI:** 10.1038/s41467-019-13112-6

**Published:** 2019-11-15

**Authors:** Jonathan M. Taylor, Carl J. Nelson, Finnius A. Bruton, Aryan Kaveh, Charlotte Buckley, Carl S. Tucker, Adriano G. Rossi, John J. Mullins, Martin A. Denvir

**Affiliations:** 1 School of Physics and Astronomy, University of Glasgow, Glasgow, UK; 2 British Heart Foundation Centre for Cardiovascular Science, Queen’s Medical Research Institute, University of Edinburgh, Edinburgh, UK; 3 Centre for Inflammation Research, University of Edinburgh Medical School, Teviot Place, Edinburgh, EH8 9AG UK

**Keywords:** Time-lapse imaging, Light-sheet microscopy, Software, Light-sheet microscopy

## Abstract

Three-dimensional fluorescence time-lapse imaging of the beating heart is extremely challenging, due to the heart’s constant motion and a need to avoid pharmacological or phototoxic damage. Although real-time triggered imaging can computationally “freeze” the heart for 3D imaging, no previous algorithm has been able to maintain phase-lock across developmental timescales. We report a new algorithm capable of maintaining day-long phase-lock, permitting routine acquisition of synchronised 3D + time video time-lapse datasets of the beating zebrafish heart. This approach has enabled us for the first time to directly observe detailed developmental and cellular processes in the beating heart, revealing the dynamics of the immune response to injury and witnessing intriguing proliferative events that challenge the established literature on cardiac trabeculation. Our approach opens up exciting new opportunities for direct time-lapse imaging studies over a 24-hour time course, to understand the cellular mechanisms underlying cardiac development, repair and regeneration.

## Abstract (web summary)

Imaging heart development is challenging due to constant tissue movement and changing physical landmarks. Here the authors present an algorithm capable of maintaining phase-locked imaging throughout a 24 hour timespan, enabling long term timelapse imaging studies of zebrafish heart development, repair and regeneration.

## Introduction

The ability to image an embryo at a cellular and subcellular level is crucial for understanding the dynamic processes and interactions underpinning development^1–6^. The ideal imaging system would acquire high-resolution three-dimensional (3D) images of specific organs and structures continuously over relevant time periods, typically hours or days, without causing tissue damage or interfering with the anatomical or physiological state of the organism. While light sheet microscopy has emerged as a valuable in vivo imaging solution to this challenge^7,8^, imaging the complex 3D structure of the beating heart has additional challenges due to its constant cyclic motion at rates of 120–180 beats per minute. This problem can in principle be overcome by using synchronisation techniques to acquire 3D images free from motion artefacts^9–11^. However, to date, only some aspects of this problem have been solved at any one time. Imaging of the developing fish and chick heart has been previously reported for: single 3D snapshots at selected intervals^11^, 3D images of a single cardiac cycle^12,13^, sampling of a limited number of time points during development^9,14^, and 2D time-lapse video^15^. Temporarily stopping the heart using high-dose anaesthetic has also been used to acquire 3D image sequences of the heart^16^ but such approaches can significantly alter the physiological state of the heart^17^ and the wider embryo^18^, particularly when performed repeatedly in the same embryo at multiple timepoints. No method exists that can sustain day-long synchronised 3D imaging at the sufficiently short time-lapse intervals required to reliably track mobile cells and visualise specific cellular and subcellular events in the unperturbed heart.

The challenges of time-lapse 3D cardiac imaging can be understood on three key timescales. Within one cardiac cycle there are rapid changes in shape and size of the heart over less than a second. From one cardiac cycle to the next the heart adopts a highly repeatable sequence of shapes, although there can be subtle changes in rhythm from one beat to the next. However, on longer timescales dramatic structural and functional changes occur at a cellular and whole-organ level, especially during development where the heart undergoes significant morphological changes. It is important to be able to maintain stable imaging over all of these timescales in order to fully understand the biological processes of interest. For example, cell shape changes and immune cell migration takes place over minutes, while cell proliferation and acute inflammatory responses occur over hours. At a whole-heart level, developmental processes such as cardiac morphogenesis take 2–3 days to complete, and injury-associated inflammation also takes several days to resolve. Synchronisation algorithms for day-long imaging must eliminate intra-beat motion, visualise inter-beat motion, and be tolerant of morphological changes on developmental timescales. It is also crucial that they do not require an increased phototoxic laser exposure compared to time-lapse imaging in non-moving tissue.

**Fig. 1:**
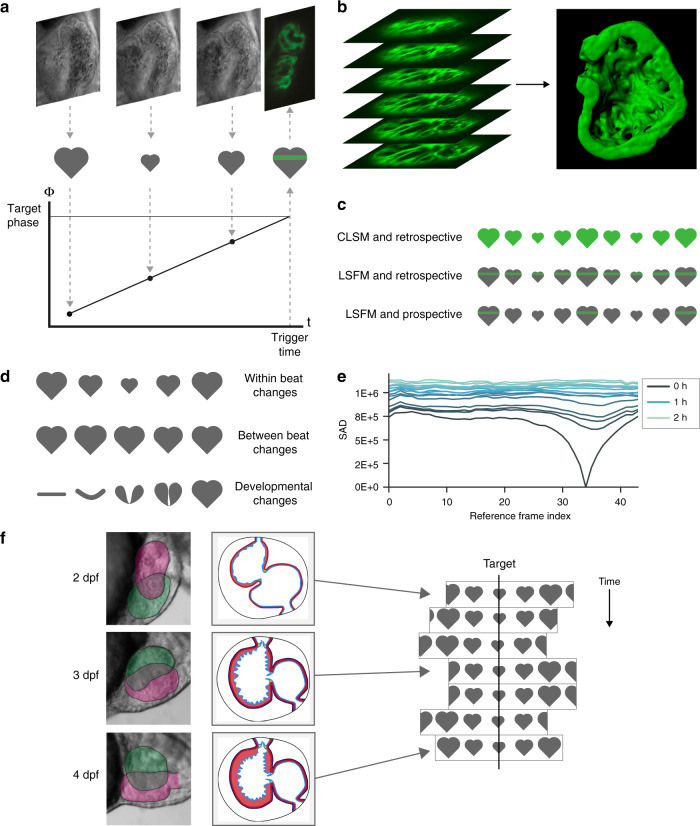
Developmental changes in cardiac morphology prevent day-long time-lapse imaging with existing algorithms. **a** In prospective optical gating, brightfield images (greyscale) are acquired and analysed in real time to assign a phase (temporal position in the cardiac cycle) to each frame. Forward prediction is then used to accurately trigger acquisition of one synchronised fluorescence image (green) at a user-defined target phase. **b** The sample is scanned through the light sheet to generate a synchronised 3D $z$-stack. **c** The move from confocal laser scanning microscopy (CLSM; upper) to light sheet fluorescence microscopy (LSFM; middle) reduces phototoxic effects by several orders of magnitude by limiting excitation in space. Similarly, the move from retrospective (upper and middle) to prospective gating (lower) reduces phototoxic effects yet further by limiting excitation in time. **d** Prospective optical gating relies on periodic changes within heartbeats (upper) and is able to cope with small changes between heartbeats (middle). However, phase-lock cannot be maintained over developmental time scales, because the heart undergoes drastic morphological changes (lower). **e** These changes mean that image-based similarity metrics are unable to match new brightfield images against the reference heartbeat image sequence recorded at the start of the experiment. **f** A new “smart microscope” is required that can maintain phase-lock in the face of these drastic changes in cardiac morphology over the course of day-long time-lapse imaging

We have previously demonstrated prospective optical gating in the beating zebrafish heart^10,11^, stroboscopically building up a synchronised 3D $z$-stack of the heart at a single time point without motion artefacts (i.e. the imaging is phase-locked) such that the heart is at exactly the same phase in the cardiac cycle when every plane is imaged (Fig. 1a, b, Supplementary Video 1). In contrast to alternative retrospective optical gating approaches requiring several orders of magnitude more fluorescence images to be acquired^9^, we only needed to acquire one single fluorescence image per $z$-plane per timepoint. Thus, in the same way that light sheet microscopy eliminates redundant excitation of fluorescence in the spatial domain, the philosophy of our prospective approach is to eliminate redundant excitation in the time domain (Fig. 1c). However, our previous algorithm relied on the assumption of quasi-periodic, stereotypical motion across all cardiac cycles in the experiment. This assumption is drastically violated on developmental timescales, as the heart changes in position, size and shape: correct phase-lock is lost within approximately 1 h (Fig. 1d, e), making it impossible until now to maintain live time-lapse imaging of the heart across developmental timescales.

We have now developed new algorithms to overcome this barrier and achieve day-long, synchronised 3D time-lapse imaging in the beating heart, involving both algorithmic and optical advances. By implementing these on a light sheet fluorescence microscope (Supplementary Fig. 1) we have been able, for the first time, to image a live, physiologically unperturbed, beating zebrafish heart over 24 h. This novel capability has allowed us to track cardiac developmental morphogenesis and patterning with high temporal and spatial resolution in 3D, and track individual motile cells, including cardiomyocytes and inflammatory cells associated with heart development and heart injury for the first time. The established understanding of zebrafish heart development on a cellular level has been built up without the benefit of direct video time-lapse imaging; we will reveal how studies enabled by our new adaptive prospective optical gating are already yielding evidence that reconciles and extends recent biological literature on cardiac development.

## Results

### Adaptive prospective optical gating for longitudinal imaging

No existing synchronisation algorithms can maintain day-long time-lapse imaging. Existing prospective synchronisation algorithms fail completely within approximately 1 h due to the changing appearance of the heart (Fig. 1d–f; Supplementary Video 2) and long-term sample drift. As we show later, postacquisition strategies are not suitable due to their phototoxic and photobleaching effects, and furthermore published postacquisition algorithms cannot even be applied across these timescales without the new developments we report here. We therefore developed a new adaptive prospective optical gating algorithm suitable for automated day-long time-lapse imaging.

**Fig. 2:**
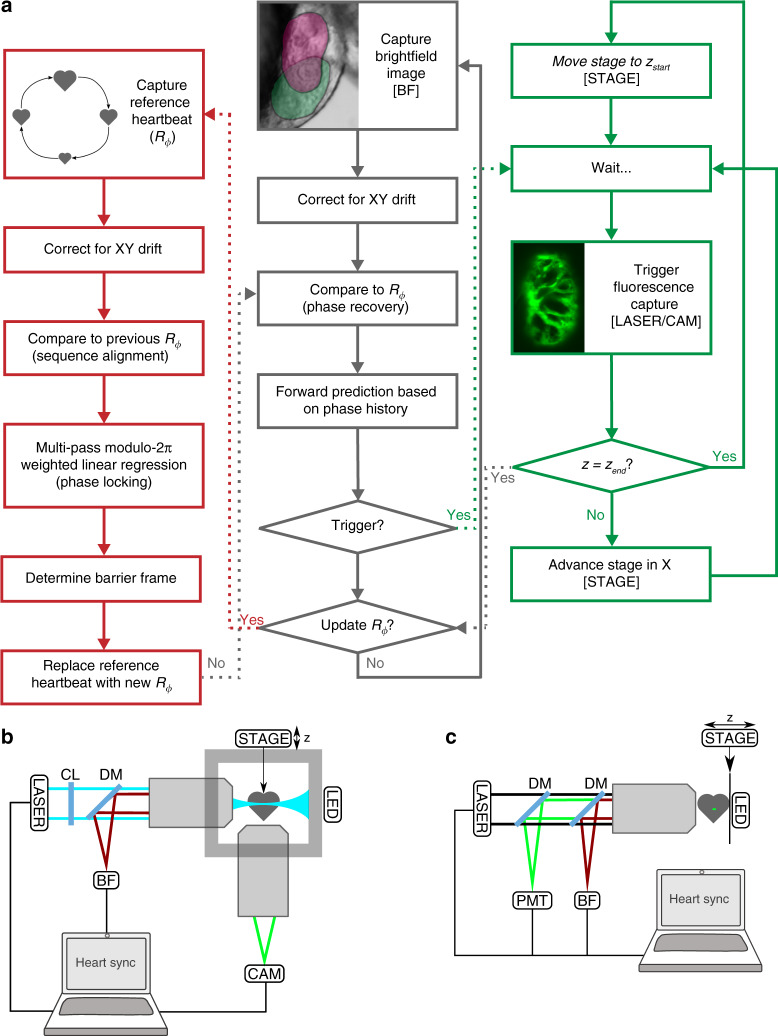
Adaptive prospective optical gating permits day-long, phase-locked cardiac imaging. **a** Summary flow chart for adaptive prospective optical gating. Each column represents a separate thread running concurrently on the computer. The core prospective optical gating algorithm (centre, grey) identifies the current cardiac phase using the brightfield channel, and computes the correct future trigger time. The day-long phase locking algorithm (left, red) enables the system to cope with developmental-scale changes in cardiac morphology and size. The optical gating algorithms are integrated with fluorescence $z$-stack acquisition and stage movement (right, green). Square brackets refer to hardware components in the schematic diagrams below. **b**, **c** Simplified schematics of the custom light sheet microscope (**b**) and commercial two-photon microscope (**c**) used in this paper, highlighting the key components for adaptive prospective optical gating. Fluorescence (excited by LASER) is imaged onto a triggerable scientific camera (CAM) or photomultiplier (PMT). An infrared light source (LED) provides illumination for a brightfield camera (BF); in **b** this receives light collected through the laser launch objective, for reasons explained in the main text. A full optical diagram for **b** can be found in Supplementary Fig. 1. Other components: STAGE motorised *z*-stage; CL cylindrical lens; DM dichroic mirror to separate different light wavelengths

Our algorithm is based around a new multi-pass sequence alignment algorithm, to enable us to maintain high-precision beat-to-beat synchronisation in spite of the developmental changes occurring in the organism (Fig. 2a). Prospective gating computationally eliminates intra-beat motion of the cardiac tissue, allowing inter-beat changes (e.g. immune cell migration) to be observed, but phase-lock is lost as the appearance of the heart changes over the course of embryonic development. At regular intervals (typically after each $z$ stack) we therefore “refresh" our reference brightfield image sequence (i.e. acquire a new sequence, which is then used for prospective optical gating to assign a cardiac phase to every subsequent image we acquire). To maintain a fixed phase-lock in spite of this refresh we perform a sequence alignment operation (in the terminology of retrospective optical gating algorithms^12^) to identify the location in the new image sequence that matches the target synchronisation phase in the previous reference image sequence (Fig. 1f; Supplementary Video 3). However, we discovered that published retrospective algorithms are unable to work with image sequences of the type required for long-term synchronisation (due to issues associated with phase-wrapping—details in Supplementary Fig. 2), so we developed our multi-pass sequence alignment algorithm to permit robust determination of the absolute shifts between video sequences and maintain phase-lock. To maintain stable phase-lock it is also essential that our algorithm compensates for the natural movement of the heart within the brightfield field of view, due to growth as well as any gradual slight drift of the sample within the mounting apparatus.

To ensure sufficient reliability and robustness for unattended day-long running we also developed an improved strategy for forward prediction in prospective gating, incorporating a biological understanding of heart rhythm variability. Our previously published prospective gating algorithms for acquiring individual $z$-stacks^10^ required human fine-tuning of the algorithm parameters for different phases in the cardiac cycle, and different ages of fish. We now incorporate an automated understanding of where in the reference image sequence the refractory period between heartbeats is located. Since this interval varies slightly in duration from one beat to the next, it poses a challenge for forward prediction to anticipate the correct time to send an electrical synchronisation trigger. We exploit this knowledge to ensure that our linear fitting for forward prediction does not attempt to fit across this variable interval. Thus we ensure synchronisation can be established reliably by the user at the start of an overnight time-lapse experiment and, most importantly, that performance is maintained throughout the whole experiment in spite of considerable changes in heart rhythm and appearance, for instance due to embryonic development or as a consequence of injury.

Finally, to overcome limitations on available $z$ scan range in our previous work, we adopted a different optical strategy for brightfield imaging which enabled us to image both chambers of the heart in arbitrary orientation, and allowed extra room for growth and movement on developmental timescales, as well as capturing active cells such as immune cells in the external vicinity of the heart. All prior published work has acquired brightfield images through the fluorescence imaging objective. In the present manuscript we instead use side-view brightfield imaging through the laser launch objective in the light sheet microscope (Fig. 2b), which means that during a $z$ scan the brightfield images do not change in focus, and instead the images are simply translated sideways. This alternative strategy was essential to provide the necessary $z$ scan range, as well as being much less cumbersome than our previously published approach involving motor-driven tube lenses^11^. To avoid having to considerably increase the region-of-interest processed by our algorithms (to accommodate this sideways translation) we implemented a real-time-adaptive camera region-of-interest for this brightfield camera (Supplementary Note 1).

Our algorithms ensure that phototoxicity is minimised and dataset sizes are kept manageable, enabling us to perform detailed assessment of structural changes in the developing heart, and visualise cellular and subcellular behaviour, near to continuously, over the course of hours and days.

### Capturing developmental changes in the living zebrafish heart

**Fig. 3:**
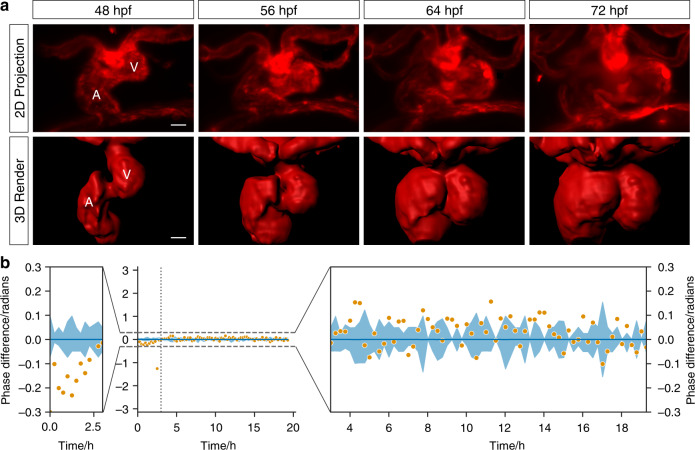
Phase-locked, day-long cardiac time-lapse imaging of cardiac morphogenesis. **a** The process of cardiac looping, previously observed by histological studies at significantly lower temporal resolution, is now seen in direct 3D video detail (48–72 hpf, at 300 s intervals). The endothelium lining blood vessels and heart chambers is seen during completion of cardiac looping (red—transgene *flk1*:mCherry). Selected timepoints shown as maximum intensity projections (MIPs) of $z$-stacks and 3D render (from Supplementary Videos 4 and 5). V ventricle; A atrium. **b** Adaptive prospective optical gating algorithm (orange) phase-lock performance is compared against human judgements of best-matching frames (blue; line represents mean and shaded area represents the standard deviation). Viewing over the full $2\pi$ range (middle) confirms the high precision and accuracy of the gating. Zoomed details (left and right) reveal a minor residual drift in phase during the early time period in which the heart underwent the most dramatic changes in shape and position, but otherwise very high stability

To validate both the technical performance of our method and its biological applicability, we imaged cardiac looping, a stage of cardiac morphogenesis that, although well-characterised, has never before been imaged in its entirety in 3D due to the lack of suitable imaging technology. Cardiac looping commences at approximately 24 h postfertilisation (hpf) when the zebrafish heart tube elongates asymmetrically, and by 36 hpf the future ventricle migrates ventrally in relation to the atrium^19,20^. By 48 hpf, the looped zebrafish heart appears S-shaped and undergoes more subtle changes until both chambers are morphologically distinguishable^19,21^. Using our adaptive prospective optical gating software, we have shown that we can directly witness these changes in 3D time-lapse video (Fig. 3a; Supplementary Videos 4 and 5), acquiring a $z$-stack every 5 min. We observed the pinching of the atrioventricular canal region, followed by expansion of the outer curvature of the atrium and finally the ventricle. Over the course of this enlargement we measured a 300% increase in endocardial chamber volume between 48 and 72 hpf. To our knowledge, this is the first reported 3D time-lapse movie detailing cardiac morphogenesis of a live beating zebrafish embryonic heart.

Cardiac looping is one of the most challenging scenarios for time-lapse gated imaging, due to the dramatic structural and functional changes involved. To explore the accuracy of our optical gating algorithms we compared our algorithm’s phase-lock at a specified phase in the cardiac cycle against a human-based manual annotation over the course of a time-lapse experiment (Fig. 3b), since no previous automated method exists for comparison. For the majority of the experiment there was a strong agreement between the target frame identified by our fully automated software algorithms and that chosen manually by five human experts (who were shown high-quality images of the heart and asked to search for a fixed target phase point in the cardiac cycle—see Online Methods). For the majority of the time-lapse experiment the small variance between our algorithms and the human mean (${\sigma }^{2}=0.0035$ radians) was slightly smaller than that between individual experts (${\sigma }^{2}=0.0050$ radians), in spite of significant developmental changes in cardiac shape and size. The similar values for variance confirm that the human experts cannot be considered a gold standard: variation between human individuals is on a similar level to any potential difference between algorithm and human assessment. In the first $\sim$3 h of the experiment there was a slight but distinct disagreement between the frames selected by the algorithm and those selected by the humans. This time-frame corresponded to the end of cardiac looping, where the changes in appearance of the heart mean that there is an element of subjective judgement even in determining whether two phase points should be considered equivalent. Nevertheless, even during this time-frame, the stack-to-stack precision of the synchronisation algorithm (the most important quality criterion for applications such as cell tracking) remained within the range of human variance.

### Tracking and assessing inflammatory cell behaviour

The ability to track immune cells and image their physical interactions in tissues is essential for understanding any immune response to cardiac injury. However, cardiac motion artefacts dominate completely over immune cell motions, precluding attempts to perform live imaging at a cellular level in the heart. Our ability to computationally freeze the gross motion of the heart over day-long study periods has now made it possible to directly image and study, in 3D, immune cell interactions on, and with, the beating heart.

**Fig. 4:**
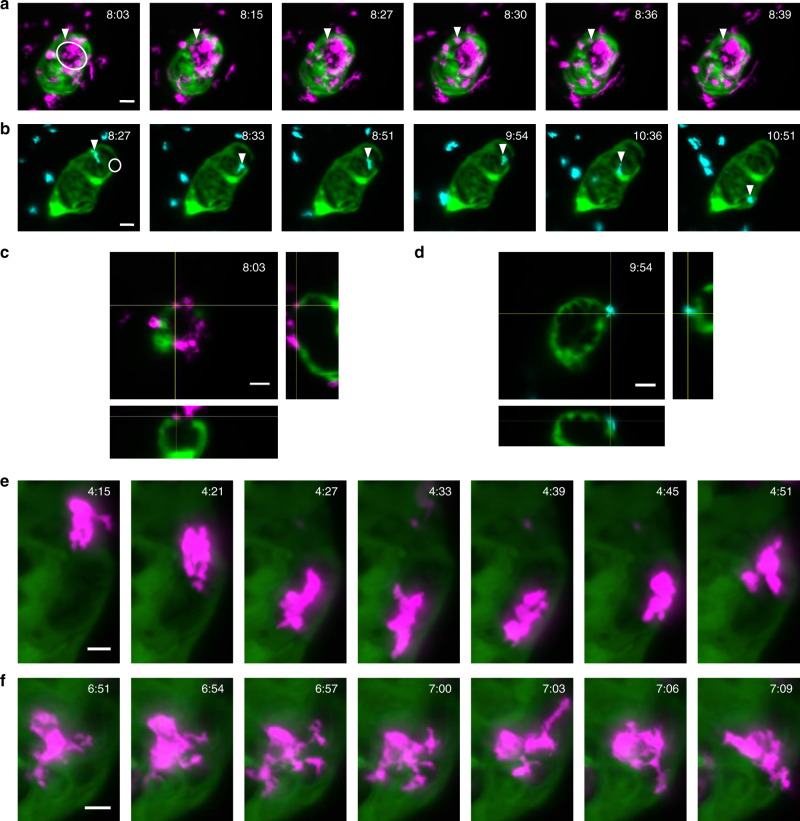
Sustained beating-heart time-lapse imaging of immune cell responses to cardiac injury. **a**, **b** Sequence of MIPs (from Supplementary Videos 6–8) showing macrophages (**a** magenta *mpeg1*:mCherry) and neutrophils (**b** cyan *mpx*:mCherry) migrating to, and interacting at, the laser wound site (white circle) on the ventricle (*myl7*:GFP, laser injury has bleached myocardial cells at the injury site). Arrowheads mark tracked immune cells interacting with the wound margins, changing shape and reverse migrating from the wound to the pericardium. **c**, **d** Orthogonal plane views co-localising the tracked macrophages (**c**) and neutrophils (**d**) on and within the myocardium at the wound site. **e**, **f** Individual macrophages are seen to undergo complex shape changes during migration. All timestamps show hours post injury (hpi). Scale bars: 30 μm (10 μm for **e**, **f**)

To demonstrate this novel capability, we subjected 72 hpf zebrafish to a cardiac injury by targeting the ventricular apex with a precise laser pulse^22^, and acquired $z$-stacks of injured hearts at 180 s intervals for approximately 24 h in macrophage and neutrophil fluorescent reporter lines (Fig. 4a, b, respectively). This allowed us to readily observe the morphology, behaviour, and velocity of macrophages and neutrophils accumulating at the injured ventricle (Supplementary videos 6–8). Neutrophils display a rounded morphology and an amoeboid mode of migration to the wound by extending pseudopods^23^. Individual neutrophils and macrophages can be observed interacting with the wounded myocardium, with orthogonal 3D views (Fig. 4c, d) centred on each tracked immune cell confirming that these cells are in direct contact with the myocardium and hence physically interacting with the wound. Tracking of specific individual neutrophils identifies their retention at the wound, distinct from other nearby neutrophils which continue to migrate within surrounding tissue. In contrast, we observe that macrophages display a range of changing morphologies from dendritic-like (Fig. 4e, f) to spherical, and appear to migrate more slowly to and from the wound site, in a mesenchymal mode of migration (Supplementary Video 6).

Exploiting this direct imaging capability, we next assessed whether our optical-gating technique could facilitate quantification of subtle immune cell behaviour at the wound site, as previously studied in non-motile tissues^24,25^ but until now impossible in the heart in vivo. Here we challenged ourselves and our imaging modality by focusing on neutrophils (known to be approximately 3$\times$ faster than macrophages^26^ and therefore more difficult to track precisely in time-lapse). Neutrophils were tracked to determine cell velocity and meandering index (displacement$\,\div\,$distance), two parameters commonly used to assess changes in migratory behaviour following injury^25,27^. The meandering index quantifies the tortuosity of a motile cell’s track. For example, when neutrophil migration is direct and linear through tissue, the meandering index is high, whereas at a wound site neutrophil migration follows a more zig-zag path and the meandering index is low. Neutrophils observed to scout the wound are distinguishable from other patrolling cells by their low meandering index, thus validating the ability of our imaging approach to extract quantitative data relating to immune cell behaviour.

Given the relatively high mean velocity of neutrophils, we demonstrated that the high temporal resolution of our adaptive prospective optical gating was essential for accurate measurement of cell behaviour metrics such as meandering index (Supplementary Fig. 3). Repeating our previous analyses using longer time intervals between $z$-stacks, for example, reveals that intrinsic information on neutrophil migratory behaviour is lost when using longer time intervals. Only with the full range of behavioural information, requiring short-interval 3D acquisition rates, is it possible to make a correct quantitative assessment of the different subpopulations of immune cells migrating in the vicinity of the heart.

### Minimal photo-injury and photobleaching

A key advantage of our adaptive prospective optical gating approach is that it minimises the number of fluorescence images acquired, and hence minimises the deleterious effects of laser exposure on the tissue (see refs. ^28,29^ for recent discussions). Light sheet fluorescence microscopy is well known for its ability to reduce photobleaching and photodamage by limiting fluorescence excitation to only the spatial plane(s) of interest^30^; adaptive prospective optical gating further limits fluorescence excitation to only the times (i.e. cardiac phase) of interest.

**Fig. 5:**
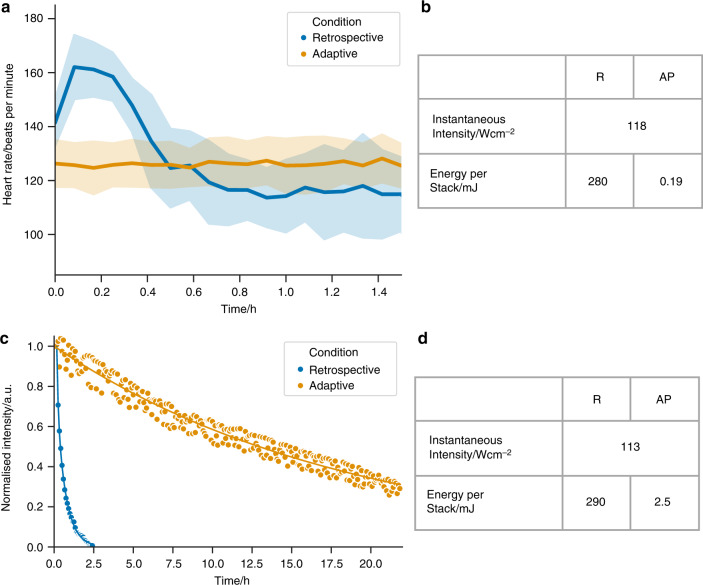
Adaptive prospective optical gating minimises cardiac photo-injury and photobleaching. **a** Acquisition of multiple fluorescent *z*-stacks in a zebrafish heart (transgene *myl7*:GFP; 3 dpf) using a retrospective optical gating protocol (blue; mean (line) with standard deviation (band)) causes an increase, followed by a fall, in heart rate over a 1.6 h imaging period ($n=6$ fish). Meanwhile 1.6 h of continuous image acquisition using our adaptive prospective optically-gated protocol (orange; mean (line) with standard deviation (band)), with stacks acquired every 5 min, causes no change in heart rate ($n=5$ fish). Plots for individual fish can be seen in Supplementary Fig. 4. **b**, **d** The high energy per stack required for retrospective gating (R) is significantly reduced with adaptive prospective (AP) gating. **c** When exposed to light levels required for retrospective gating (using typical parameters reported in the literature, and again acquiring $z$-stacks at 5 min intervals), the fluorescence signal from a bleach-susceptible fluorophore (*myl7*:mKate-CAAX) is halved after only 3 $z$-stacks (blue). In contrast, adaptive prospective optical gating achieves 150 $z$-stacks before showing an equivalent fall in fluorescence intensity (orange). Time course shown in Supplementary Video 9. Data show one representative fish for each condition

To quantify the physiological impact of our own strategy against that of established retrospective gating strategies, we used heart rate as a measure of induced photo-injury (see Online Methods). For fish exposed to 2 h of retrospectively gated imaging at 5 min intervals, we observed an initial increase followed by a significant reduction in heart rate (Fig. 5a). We conjecture that this is due to an initial heating, causing an increase in heart rate, followed by a patho-physiological response, resulting in bradycardia, due to photodamage and phototoxicity to the heart. These effects were accompanied by changes in heart rhythm ($n=5$ of $6$ fish) and reduction in ventricle ejection fraction (Supplementary Video 9), both further signs of physiological impact. In contrast, time-lapse imaging using our method at 5 min intervals caused no significant change in heart rate over a similar time period (Fig. 5a).

The reduced laser dose with our method also has the important effect of reducing the rate of photobleaching. This is particularly important for easily bleached fluorophores and for the low endogenous expression levels obtained using CRISPR-Cas9 technology^31^. We demonstrated this by acquiring 3D time-lapse fluorescence images using two alternative imaging protocols (Fig. 5c, Supplementary Video 10). The first protocol, representing a retrospective optical gating approach, causes rapid and marked bleaching of mKate emission from cardiomyocyte cell membranes and the signal soon becomes indistinguishable from background. In contrast, our imaging protocol causes only mild and gradual photobleaching, and can continue to capture 3D images for over 24 h. Direct reduction in bleaching rate is due to a combination of the stroboscopic nature of imaging^32^ and the reduced total laser dose^33^. Additionally, a reduction in photo-injury in the tissue may minimise the oxidative rate of fluorescent molecules, as previously seen in in vitro samples^34^, thus indirectly reducing photobleaching still further.

### Novel insights into cell proliferation and heart development

**Fig. 6:**
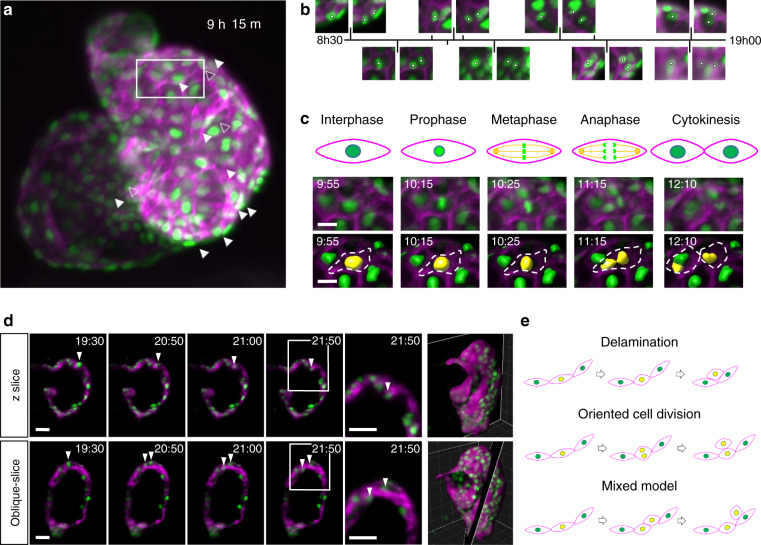
Mapping cardiomyocyte migration and cell division during myocardial trabeculation. **a** Representative MIP from a 24 h time-lapse of a 72–96 hpf zebrafish (Supplementary Video 10). Image shows cardiomyocyte nuclei (green—transgene *myl7:h2b-gfp*) and cardiomyocyte cell membranes (magenta—transgene *myl7:mKate-CAAX*), with strongest signal expressed in the ventricle. Nuclei of cardiomyocytes which will later divide are indicated by white arrowheads (open-arrowheads for less-obvious divisions near the posterior wall of the heart). Region-of interest for **c** is indicated with white box. **b** Detail from time-lapse: pairs of images plotted on a timeline, illustrating selected cardiomyocytes shortly before (left) and after (right) cell division; nuclei indicated with white circles. **c** A selected cardiomyocyte division clearly exhibits key stages of mitosis. Mitotic stages (schematic: top row) are visible at selected timepoints from the time-lapse sequence (MIPs: middle row), and the nuclear volume changes and karyokinesis typical of cell division can be seen (dividing nucleus surface-rendered in yellow: bottom row). **d** A dividing cardiomyocyte nucleus is shown in the acquisition plane ($z$-slice, top row) and in the plane of division (oblique slice, bottom row) across two and a half hours of imaging with 5 min time-lapse resolution. White arrowheads highlight the observation of two daughter cardiomyocyte nuclei in the oblique-slice, which would have been missed in the $z$-slice or 2D imaging. The final time point is shown zoomed-in to highlight the migration of the two daughter cells across the time period. Whole rendered heart ventricle (magenta) with rendered nuclei (green) indicate the $z$-slice and oblique-slice views used. **e** Schematic illustrating three models of cardiac trabecular initiation: delamination model (top), cell division model (middle) and mixed model (bottom). Delaminating cardiomyocytes are indicated by a yellow nucleus. Scale bars: 30 μm (10 μm for **c**)

Our new platform is allowing us to study complex developmental biological questions. Our ability to capture phase-locked $z$-stacks at time-lapse intervals as close as 3 min apart allowed us to routinely map all cell division events in the beating heart (Fig. 6a, b), capturing the archetypal stages of mitotic cell division at both the subcellular and cellular level (Fig. 6c). In examining such events we observed novel behaviour of dividing cardiomyocytes, involving rapid migration of daughter cells following division. We conjecture that this may be a consequence of the mechanical forces acting on newly formed cardiomyocytes in the beating heart, before they have formed strong cell–cell adhesions. Such events could not have been observed previously where the heart had to be arrested to acquire each $z$-stack. Interestingly, we observed cell division of cardiomyocytes occurring at a rate of roughly 1 per hour between 72 and 96 hpf (Fig. 6b), a higher rate of cell division than reported previously [ref. ^6^, Fig. 1]. We hypothesise that this is a consequence of the more physiological conditions maintained by using our gating system.

An important early developmental process where cell division may play a role is trabeculation, whereby multicellular luminal projections of cardiomyocytes are formed as the heart matures (Supplementary Fig. 5a shows trabeculation at the tissue level in the same zebrafish heart during three consecutive days). Two principal mechanisms have been proposed for initiation of trabeculation^35^. The first is referred to as delamination, where cardiomyocytes are physically squeezed-out luminally from the cortical myocardium layer without a cell division event. The second is referred to as orientated cell division where cortical cardiomyocytes divide perpendicular to the ventricular myocardium. Currently, delamination is suggested to be the sole mechanism of trabeculation initiation in zebrafish^35^.

Detailed observation of cardiomyocyte cell division and migration enabled us to study cardiac trabeculation initiation (Fig. 6a, b; Supplementary Video 11). Using our imaging system we were able to assess whether orientated cell division could also contribute to trabeculation. We observed cell division events and ventricular cardiomyocyte migration occurring both parallel and perpendicular to the abluminal-lumen axis (Supplementary Fig. 5b). Interestingly, tracking the migration of one such cardiomyocyte in the original plane of acquisition (Fig. 6d) initially indicated that it delaminates from the cortical layer without dividing, and only on closer examination in 3D (re-slicing the full $z$-stack along additional planes) was it revealed that this cardiomyocyte actually undergoes cell division, parallel to the abluminal-lumen axis, while also delaminating perpendicularly to give rise to a trabecular projection into the lumen. These observations appear to suggest what we call a “mixed model" for early trabeculae formation in the ventricle, whereby cardiomyocyte division is immediately followed by luminal migration of a daughter cell (Fig. 6e). It is evident that despite the range of genetic, histological, and imaging techniques available to the community at present, all have failed to capture this biology until now. Combining our high-resolution 4D imaging system with cell signalling reporter lines will permit future investigation of the underlying molecular control of trabeculation; candidates include notch and neuregulin^36,37^.

Mitosis of cardiomyocytes has previously been studied in situ using labour-intensive methods that have pushed the limits of what was possible at the time, by imaging after cardiac arrest^5,35^ or using high-speed 2D video imaging^6^. However, these approaches have a number of critical limitations. Firstly, repeated pharmacological arrest and restarting of the heart is liable to impact cellular events, and excessive phototoxic laser exposure will do the same^28^. Secondly, when imaging during cardiac arrest, the heart is not subject to its usual mechanical forces, thus potentially missing key observations linked to stress and strain within the myocardium. Our approach eliminates all these issues. The rapid rate with which daughter cells separate in three dimensions means that our use of short-interval 3D time-lapse imaging was essential to capture cell division and cytokinesis events, interpret them correctly (Supplementary Fig. 5c), and reach the conclusions that we have drawn.

## Discussion

Time-lapse imaging cannot offer biological insights if the imaging procedure itself causes severe disruption to the physiology of the organism. Our method and results demonstrate for the first time how to avoid this in the heart, and indeed how to make day-long time-lapse imaging survivable at all at a biologically relevant, rapid acquisition rate. Light sheet microscopy reduces the damaging laser exposure by orders of magnitude compared to confocal imaging, but this benefit is lost when using previous methods for time-lapse beating-heart imaging in which hundreds of fluorescence images must be acquired in each plane prior to retrospective synchronisation^13^. Alternatively, repeated pharmacological arrest of the heart using high-dose anaesthetic permits artefact-free imaging, but at the cost of accumulating exposure of the heart and the whole organism to anaesthetic agent. With our new algorithms we have eliminated both these obstacles to survivable and physiologically realistic long-term imaging. Our quantitative measurements of heart rate as a measure of phototoxic effects are, to our knowledge, the first reported longitudinal quantifications of the patho-physiological impact of fluorescence imaging in vivo in a vertebrate. This is an increasingly important consideration in the context of ethical approval for studies in older (and legally protected) specimens. We anticipate that our results will not only spark further studies quantifying the impact of imaging, but also inspire further development and validation of suitable in vivo phototoxicity assays.

For the developmental and cell migration studies presented here, only images at a single heart phase need be acquired, minimising phototoxic laser exposure. Conversely, retrospective gating strategies do offer the freedom to make post hoc decisions to reconstruct images from any or all phases of the cardiac cycle, which is advantageous for studies of cardiac dynamics^38^. We note that if additional phases in the cardiac cycle are required, for example for ejection fraction measurement, blood flow mapping^39^, or studies of heart wall dynamics, then our approach can be readily adapted to trigger images at multiple precise phases throughout the cardiac cycle (or indeed to assign phases in real time to a continuously acquired stream of fluorescence images^39^). Our multi-modality approach, using brightfield images to trigger synchronised acquisition of the fluorescence image, separates the conflicting requirements for fluorescence imaging (tissue-specific labelling, which may be highly sparse; $z$-scanning; $z$-sectioning while minimising photodamage) and heart-synchronisation (phase-diverse periodic signal; invariance during $z$-scan), offering additional flexibility to optimise future microscope designs for cardiac imaging. Recent advances in fast volume imaging using custom-built adaptations of light sheet microscopes^40–42^ may soon permit direct, synchronisation-free snapshot 3D imaging of the heart. Even as these techniques mature, for time-lapse imaging our real-time triggering capability will still remain essential for maintaining phase-lock throughout repeated time-lapse 3D image acquisitions. Using a brightfield synchronisation source also has other potential uses such as fusing and phase-registering multiple fluorescence channels with sparsely labelled (and less clearly periodic) structures^39^, or acquiring synchronised images in multi-acquisition modalities such as structured illumination microscopy.

Our method brings two important practical advantages for an experimental campaign: (i) it allows unsupervised day-long imaging, without the need for human intervention at every imaging timepoint; (ii) it substantially reduces data file sizes, eliminating the considerable challenges of storing and processing multi-TB datasets. For example, if a retrospective gating approach had been used for the experiments in Fig. 3, the raw data for one time-lapse would have occupied at least 16 TB (assuming 200 video frames per slice^13^), and would have required days of postprocessing time. In contrast, our datasets occupy only 80 GB of storage space. These relatively modestly sized datasets can also be accessed and reviewed by the user through the course of the experiment, paving the way for future developments such as user-initiated or automated responses to unexpected or atypical biological observations during imaging experiments.

We have demonstrated that our optical gating system can be used to clarify complex biological patterning such as the cellular processes underlying myocardial trabeculation. Previous studies have used genetic tools to stochastically label cardiomyocytes with different fluorophores prior to trabeculation^35,43^. These studies reported that adjacent cortical-trabecular cardiomyocytes rarely share the same fluorophore, implying that orientated cell division does not contribute to trabeculation. However, these studies relied on analysis of single optical and histological slices of the heart and so may have missed daughter cells that have migrated away from each other out of plane. Our own observations have shown that in a number of instances cardiomyocyte cell division was followed by immediate perpendicular migration of a daughter cardiomyocyte. Coupling delamination to proliferation is a plausible strategy for trabeculation: not only would this maintain cortical cardiomyocyte number, but the disassembly of sarcomeres (known to occur prior to proliferation) may also liberate the cell for delamination. We have demonstrated that such events can be missed in studies where time-lapse imaging was acquired only in a single plane, potentially underestimating the contribution of dividing cortical cardiomyocytes to pioneer trabecular cardiomyocytes^5,6,35,43^. Our insight was only possible due to the high spatio-temporal resolution of our optically-gated imaging system.

Our observations of inflammatory cell interactions following heart laser injury also highlight the value of our method for 3D time-lapse imaging. The ability to track individual, rapidly moving inflammatory cells on the injured heart provided novel insights into neutrophil migratory behaviour on the beating heart. There is a clear potential to study pharmacological and genetic manipulation of these aspects of neutrophil migratory behaviour at the cardiac injury site. Similarly, changes in macrophage cell morphology were observed using our system, highlighting the potential to study morphological changes in heart-associated macrophages in vivo. Linking these shape changes with specific molecular phenotypes could provide new insights into distinct macrophage subpopulations in the injured heart.

Our method is equally applicable to embryos of other species such as fly, chick or mouse. It can also be applied to other microscope modalities, such as two-photon or confocal, simply requiring access to a brightfield image channel and the ability to trigger image acquisition. Our synchronisation software can run independently of the existing microscope control software, avoiding any need for complex integration at software level. We have successfully integrated our system with a commercial two-photon microscope (Fig. 2c; Supplementary Fig. 6) with a small degree of cooperation from the manufacturers over hardware integration, and in future our system could also be integrated with commercial light sheet microscopes, as well as other groups’ custom-built microscope platforms, to equip them with real-time synchronisation capabilities.

In conclusion, we have shown that our adaptive prospective optically gated imaging system can provide high spatial and temporal resolution time-lapse 3D images of the beating heart, as demonstrated here for zebrafish. The minimal phototoxicity of our method ensures that high-quality 3D images can be reliably acquired throughout 24 h time-lapse studies, allowing us to observe cardiac morphogenesis, inflammatory cell migration and cardiomyocyte cell proliferation. This approach has the potential to provide further novel insights into morphological, cellular and subcellular processes in the beating embryonic heart in the zebrafish and other species. Over the past decade, the transition from confocal microscope to the low-photodamage regime of light sheet microscopy has opened up a whole new landscape of in vivo biological studies involving direct and routine observation of development, cell–cell interactions and whole-embryo cell fates. We anticipate that our now-routine capability to conduct day-long time-lapse beating-heart imaging experiments represents the equivalent tipping point in cardiac biology.

## Methods

### Fish husbandry and preparation for imaging

Zebrafish husbandry, embryo collection and maintenance were performed according to accepted standard operating procedures^44^ and in accordance with the Animals (Scientific Procedures) Act 1986 in a UK Home Office-approved establishment. All experiments were performed on animals aged less than 120 h postfertilisation. Project licence approval for the maintenance of genetically modified lines was given by the UK Home Office and The University of Edinburgh Animal Welfare and Ethical Review Board.

Transgenic zebrafish lines used for imaging are as follows; Tg(*myl7:eGFP*^twu26^)^45^, Tg(*kdrl:mCherry*^ci5^)^46^, Tg(*mpx:mCherry*^uwm7^)^47^, Tg(*mpeg1:mCherry*)^48^, Tg(*myl7:h2b-GFP*^zf52^)^13^, Tg(*myl7:mKate-CAAX*^sd11^)^49^. Adults were day-crossed as appropriate to yield desired combinations of transgenes in embryos. Embryos were treated with phenylthiourea (Fisher Scientific) at 7 hpf to inhibit pigment formation and enhance image clarity. Zebrafish were embedded in 0.5% low-melting-point agarose inside FEP tubes (Adtech Polymer Engineering), which was essential to minimise drift whilst still allowing growth during long-term imaging. Fish were used only once for a time-lapse imaging experiment, and any repeats shown come from distinct individuals. All experimental procedures were performed at room temperature (23 °C).

### Light sheet microscope set-up

Our custom-built selective plane illumination microscope (SPIM) is optimised for simultaneous multi-channel light sheet fluorescence imaging of zebrafish (Supplementary Fig. 1). The detection arm is based around a Nikon Plan Fluorite ×16/0.8 NA objective lens with ultra-flat dichroic mirrors (Chroma T495lpxr-UF2, T550lpxr-UF2, T700spxr-UF2) separating red/green/blue fluorescence channels, and a near-IR, non-visible brightfield channel (which is not routinely used—see below). Fluorescence images are acquired using QIClick CCD cameras (QImaging) and fluorescence emission filters (Thorlabs MF479-40, MF525-39, MF630-69). Rather than acquiring our brightfield images through the 0.8 NA imaging objective, as might be expected, we acquire these by imaging through the light sheet launch objective (for reasons explained in Supplementary Note 1). A 650 nm shortpass dichroic mirror (Edmund Optics) serves to pick off the collected brightfield light, which is imaged onto a Prosilica GS650 CCD camera (Allied Vision).

Laser excitation is at 488 and 561 nm, using a Versalase laser system (Vortran) with single-mode fibre delivery. Brightfield illumination is via a light emitting diode at 750 nm (near infrared, non-visible). The light sheet (measured full-width-at-half-maximum 2.4 μm) is formed using a cylindrical lens and a ×10/0.3NA objective lens (Nikon). Shadow effects are minimised using an mSPIM configuration^50^ using a 4 kHz resonant scan mirror (SC-30, Electro-optical Products Corporation) to modulate the in-plane propagation angle of the light sheet, over the course of each individual image exposure. Laser pulsing and camera triggering is coordinated using a custom-built electronic system based around a StartKit microcontroller board (XMOS; software and hardware details in Supplementary Note 2) which also serves to define a universal timebase for our synchronisation analysis. The sample is positioned and scanned using a combination of manual micrometre stages and motor-driven stages (M-111.1DG, Physik Instrumente).

Image acquisition parameters used for all experiments can be found in Supplementary Table 3.

### Real-time, prospective optical gating

Real-time, prospective optical gating^10,11^ is used to trigger capture of fluorescence images at a user-selected target phase in the heart’s natural cycle. This approach allows us to computationally freeze the heart without any invasive approaches such as pharmaceuticals or pacing. Real-time, prospective optical gating relies on information captured in the brightfield channel (for which even continuous illumination is not sufficiently intense to cause harm to the specimen). First, a reference sequence of brightfield images is collected, lasting precisely one natural heart cycle. Subsequent brightfield images are correlated against this to assign them a phase. A target phase in the heartbeat is selected by the user, and all subsequent fluorescence images will be triggered at this target phase in the heartbeat.

To generate a trigger requires forward prediction of when the heart will next be at the correct point in its cardiac cycle, and for this prediction we assume that the phase evolution of the heart is locally linear. For the most part we find this to be a reasonable first approximation even in the presence of heart rate variability, but the exception is the refractory period between heartbeats, whose duration may vary from one beat to the next, especially in injured or diseased hearts. Our previous approach, in which forward prediction was achieved by a linear fit in the phase domain over a fixed time interval, required manual tuning of parameters by the user, which would depend not only on the characteristics of the individual sample but also on the chosen target phase. To sustain reliable, unsupervised performance over day-long time-lapse experiments it was therefore necessary for us to incorporate into our algorithms a knowledge of where the inter-beat refractory period occurs. We achieved this via an additional algorithm parameter, the “barrier frame" (see below), which enables us to ensure that a linear fit in the phase domain is not performed across this part of the cardiac cycle.

For each brightfield image ${I}_{t}$ (composed of pixels ${I}_{t}(x,y)$) received at the current time $t$, the following steps are taken to phase-match the heart images and forward-predict the next fluorescence trigger time:

1. The new brightfield image is windowed to correct for any in-plane ($xy$) sample motion $\Delta x,\Delta y$, as calculated from the previous-received frame (see step 6).
2. The windowed brightfield image is compared against the one-heartbeat reference image sequence ${R}_{\phi }$ to identify the current phase ${\phi }_{t}$ of the heart using a sum-of-absolute-differences metric (this metric was chosen to maximise real-time processing speed):
  1 $${\bf{s}}=\sum _{xy}\left|{I}_{t}(x,y)-{R}_{\phi }(x+\Delta x,y+\Delta y)\right|$$
  2 $${\phi }_{t}=\arg {\min }_{\phi }{\bf{s}}.$$
3. The phase ${\phi }_{t}$ is refined to sub-frame precision $\phi ^{\prime}_t$ by fitting a ‘V’ function ($s=| \phi -\phi ^{\prime}_t | +{s}_{\min }$) to the three elements of ${\bf{s}}$ in the immediate vicinity of its minimum element ${\phi }_{t}$^10^:
  3 $${\phi }_t ^{\prime}=\frac{{s}_{{\phi }_{t}-1}-{s}_{{\phi }_{t}+1}}{2(\max ({s}_{{\phi }_{t}-1},{s}_{{\phi }_{t}+1})-{s}_{{\phi }_{t}})}.$$
4. To accommodate unavoidable latencies in the system, linear forward prediction of the phase evolution (details below) is used to calculate the required triggering time for the next acquisition, i.e. the precise time at which the heart will next be at the desired target phase of its heartbeat.
5. A decision is made whether to commit to the calculated trigger time or to await a refined prediction from the next incoming brightfield image. This decision is made based upon the known processing latency (~12 ms from brightfield image exposure to programming of a trigger).
6. The measure of uniform drift $\Delta x,\Delta y$ in the $xy$ plane of the brightfield images (as used in step 1) is updated using a local search to minimise ${s}_{{\phi }_{t}}$ as a function of $\Delta x$ and $\Delta y$^11^. The new values of $\Delta x,\Delta y$ will be applied when processing the next incoming brightfield image.

If the system decides to send a trigger, the target trigger time is transmitted to the timing controller over a USB/serial link. Once the timing controller has been informed of the required trigger time, it will electrically trigger the next (synchronised) fluorescence image. Following this, the sample is moved to the next $z$-position in preparation for the next synchronised acquisition.

When assigning a phase to the current brightfield image, by comparing it to the reference sequence, nonperiodic variations (such as the locations of blood cells) may result in a situation where the best match is with the very first (or last) frame in the reference sequence. This may lead to erroneous phase values being computed. We mitigate against this by padding our reference sequence with two extra frames at either end; these extra frames are not used for the initial minimum-finding, but are available in the case where the initial minimum lies at an extremum of the reference sequence.

Forward prediction is achieved by forward-extrapolating a linear fit of the recent measured phase history as a function of time. The linear fit is performed only over the phase values for frames received since the barrier frame was last passed, to ensure the linear fit is restricted to a regime where it represents a good approximation for the temporal evolution of the cardiac phase. (The fit is however always performed over a minimum of three datapoints.) The use of the barrier frame is particularly important in the case of hearts whose rhythm has been affected by injury or disease. The barrier frame is identified automatically at the start of an experiment, and while in principle the user is free to modify its value, we have found the following automated, empirical strategy for barrier frame estimation to be reliable across a range of heart orientations, ages, and states of health. Within the reference sequence, we compute the sum-of-absolute-differences between adjacent frames (yielding a simple metric ${v}_{\phi }$ for the level of motion present in each frame). We first identify the frame with minimal motion ($\arg \max {v}_{\phi }$), which is associated with the refractory period between beats. Following the refractory period the heart contracts rapidly, so we search forward in time for the first frame in which the motion metric rises to $(\max {v}_{\phi }+\min {v}_{\phi })/2$ and identify this as our barrier frame (located at the end of the refractory period).

### Adaptive prospective optical gating

Our adaptive prospective optical gating strategy requires us to be able to refresh our reference sequence, formed from video images of exactly one heartbeat, while still maintaining phase-lock at the same point in the cardiac cycle. In the terminology of the retrospective gating literature, this task is equivalent to computing the absolute alignment between all reference sequences over the course of a time-lapse experiment. However, we found that existing algorithms were not suitable for our purposes, and we had to develop a new multi-pass algorithm for absolute alignment of our reference heartbeat sequences.

Relative alignment between individual pairs of single-heartbeat reference sequences can be computed through pixel-wise temporal cross-correlation of two single-period brightfield video sequences that have been resampled to equal lengths (similar to the method used for postacquisition, retrospective optical gating^12^), as described in more detail below. However we found that use of nearest-neighbour relative alignments alone soon leads to unacceptable accumulation of errors over time, leading to loss of consistent phase-lock at a fixed point in the cardiac cycle. Previous authors have solved this problem by also considering relative alignments between sequences separated by larger time intervals, and solving the resultant over-determined system of equations to recover an improved estimate of the absolute global alignment of all sequences. However, the fact that relative alignments must be computed modulo-$2\pi$ will inevitably lead to inconsistencies in this system of equations, given that our input sequences can have arbitrary relative alignments (Supplementary Fig. 2). This led us to develop a new multi-pass algorithm^51^ to overcome this mathematical obstacle and solve for correct global sequence alignments without accumulating errors.

The algorithm proceeds as follows. First, a new brightfield reference image sequence is acquired, using image self-similarity to identify a video sequence exactly one heartbeat period long^10^. We then determine the equivalent target frame in this new reference heartbeat that matches the original, user-defined, target heart phase. This is done by cross-correlating between the original and new reference sequences as follows:

1. Each reference heartbeat (${R}_{u,\phi }(x,y)$ for timepoint $u$) is resampled (${\tilde{R}}_{u,\phi }(x,y)$) to contain a fixed, integer number of frames.
2. The relative phase shift $\Delta {\phi }_{ab}$ is computed between this new reference heartbeat, acquired at time $b$, and each of several (typically three) recent past reference heartbeats, acquired at times $a$. This shift is computed by minimising a least-squares criterion representing the similarity between relatively shifted image sequences:
  4 $$\Delta {\phi }_{ab}=\arg \min_{\Delta \phi }\sum _{xy}{\left|{\tilde{R}}_{a,\phi }(x,y)-{\tilde{R}}_{b,\phi +\Delta \phi }(x+\Delta {x}_{ab},y+\Delta {y}_{ab})\right|}^{2},$$
  where $\Delta {x}_{ab}$ and $\Delta {y}_{ab}$ represent any translation that has occurred in the image during the time elapsed between when sequences $a$ and $b$ were acquired. $\Delta {x}_{ab}$ and $\Delta {y}_{ab}$ are already known since they are tracked in real time by our prospective gating algorithms (see above).
  In fact, the above expression can be shown to be equivalent to the following criterion, based on cross-correlation along the phase axis, which we compute in Fourier space for speed:
  5 $$\Delta {\phi }_{ab}=\arg \max_{\phi }\sum _{xy}{\tilde{R}}_{a,\phi }(x,y)* {\tilde{R}}_{b,\phi }(x+\Delta {x}_{ab},y+\Delta {y}_{ab}).$$
3. Each $\Delta {\phi }_{ab}$ is refined to sub-frame precision by ‘V’ fitting (Eq. 3). To maintain acceptable precision, while ensuring a runtime compatible with real-time operation, it is essential that we do this rather than oversampling in step 1.
4. All the computed $\Delta {\phi }_{ab}$, including the relative phase shifts computed during previous reference frame refreshes, are combined into a weighted, linear least-squares regression to determine an absolute phase shift. The matrix equation to be solved has the form ${\bf{A}}{\bf{x}}={\bf{r}}$, where ${x}_{a}$ represents the absolute phase shift for sequence $a$, ${r}_{i}$ represents each computed relative phase shift $\Delta {\phi }_{ab}$, and ${A}_{a,i}=-1,{A}_{b,i}=1$ (other elements of $A$ are zero).

However, as noted above, the equation to be solved will not yield a correct global solution using least-squares matrix solver algorithms (which are not compatible with modular arithmetic). To overcome this problem, in each iteration of our multi-pass algorithm we only consider relative shifts between sequences such that $b-a\le \Delta {t}_{\max }$, where $\Delta {t}_{\max }$ represents an upper limit on the temporal distance $b-a$ being considered in that iteration. Each iteration of our new algorithm proceeds as follows:

1. Form $A^{\prime}$ by eliminating all rows from $A$ for which $b-a\,> \,\Delta {t}_{\max }$ (and form $r^{\prime}$ equivalently by discarding elements from $r$), to form a reduced equation ${\bf{A}}^{\prime} {\bf{x}}^{\prime} ={\bf{r}}^{\prime}$
2. Solve for $${\bf{x}}^{\prime}$$
3. Add or subtract multiples of $2\pi$ from each element of ${\bf{r}}$ to minimise $r-({x}_{b}^{\prime}-{x}_{a}^{\prime})$.

In the first pass of the algorithm, we only consider $\Delta {t}_{\max }=1$, i.e. temporally adjacent sequences. The effect of this is to precondition our full vector ${\bf{r}}$ of computed relative shifts, adding or subtracting multiples of $2\pi$ in such as way that a self-consistent solution for ${\bf{x}}$ is possible in conventional non-modular arithmetic. In practice, since for the present work we have found good synchronisation performance from only computing relative shifts with $\Delta {t}_{\max }$ ≤ 3, we then just performed just one subsequent pass, considering all ${\bf{r}}$. However, in cases where larger temporal distances are considered or there are high uncertainties in the individual computed relative shifts, it is preferable to perform additional passes with gradually increasing values of $\Delta {t}_{\max }$, to improve tolerance to errors.

We note that the problem we have solved with our new algorithm can also arise when using conducting postacquisition synchronisation using previously published algorithms (and hence our algorithm offers increased robustness for postacquisition as well). In practice, we presume that in previously published postacquisition experiments the problem with modular arithmetic has been largely avoided by considering only adjacent sequences, or by ensuring that adjacent sequences are separated by only a small, consistent time gap. There is absolutely no such guarantee in our more general problem of aligning time-lapse (brightfield) sequences, and it was essential that we develop this multi-pass approach in order to make our synchronisation possible at all.

### Gating on a commercial two-photon microscope

As a case study for integration with commercial microscope hardware, we applied our optical gating system to a Scientifica two-photon microscope. This required a small modification to the imaging hardware to permit simultaneous acquisition of brightfield and two-photon images, and triggering of each image acquisition using our synchronisation software and timing electronics.

The microscope has the capability for both two-photon imaging and brightfield trans-illumination in the near infrared (the latter using the same beam path as epifluorescence, which we did not use in this experiment). In a standard system the user would switch between two-photon and brightfield using a flip mirror. With the assistance of the manufacturer we replaced the flip mirror with a 800 nm shortpass dichroic mirror (Supplementary Fig. 6) such that the two-photon excitation laser is reflected at the same time as the 780 nm brightfield illumination is transmitted by the mirror. This ensures that our brightfield imaging camera, attached to the imaging port of the microscope, can be used to acquire images for input into our synchronisation algorithms, but the optical path still supports the standard two-photon image acquisition modality of the microscope. The manufacturer’s acquisition software includes a scripting capability which enabled us to sequence a $z$ scan in which the microscope waited for an external electronic trigger before acquiring the next image and advancing to the next $z$ plane. The synchronisation trigger output from our own timing controller provided this trigger input to the microscope.

When two-photon image acquisition is actively in progress, some back-reflected two-photon excitation light leaks through the shortpass dichroic mirror and momentarily saturates the brightfield camera images, but this does not cause problems for the prospective gating algorithms since it comes a considerable time before the next time we will be attempting to predict and schedule the next trigger signal. In fact, this leakage proved helpful during development since it enabled our software to identify automatically when a two-photon image acquisition had been triggered.

In this experiment we did not have control over the microscope hardware design, so it was not convenient to implement the side view or focus-correction strategies described in Supplementary Note 1. As a consequence, the focal plane of the brightfield images varied along with the focal plane of the two-photon imaging. It was therefore necessary for us to repurpose our reference sequence refresh algorithm (see previous section) to address this issue during the two-photon $z$ scan: whereas for day-long time-lapse imaging on our light sheet microscope we applied the sequence refresh algorithm between each full $z$ stack (i.e. at 3–5 min intervals), for this two-photon imaging we applied exactly the same algorithm every 3 μm within each $z$ scan. This ensured that high-quality synchronisation could be maintained even though the brightfield image appearance was changing as its focal plane changed.

### Quantitative evaluation of long-term phase-locking

To verify that our method maintains a consistent phase-lock throughout long-term time-lapse imaging, we asked experts in zebrafish heart imaging ($n=5$) to manually identify the same phase in a heartbeat (ventricular end-diastole) across a subset of the reference heartbeats used to capture the heart looping dataset in Fig. 3. Stacks were captured every 150 s using the real-time, prospective optical gating technique described above; automated long-term updating of the reference heartbeat, also described above, was carried out after every stack. Automated analysis used the (low-resolution) side-view brightfield images as usual, while human experts were shown simultaneously acquired higher-quality brightfield images acquired through the imaging objective at 0.8 NA. Without these high-quality images, it was almost impossible for the human experts to make any accurate judgement at all.

The human experts were provided with reference image sequences in a random order. These human-identified target frames were then compared to the fully automated, adaptive prospective optical gating results. In Fig. 3, neither the human nor the automated updates should be considered as the gold standard, but the close agreement between the two is further evidence that a consistent phase-lock has been maintained throughout the experiment. In the absence of a true gold standard, the human mean was treated as the zero point for each timepoint.

### Assessment of photo-injury during imaging

To assess the effect of retrospective optical gating and adaptive prospective optical gating on the physiology of the fish we compared the heart rate of fish in two different scenarios:

1. For fish undergoing 2 h of fully automated, adaptive prospective optically gated time-lapse acquisition (gated stacks acquired every 5 min).
2. For fish undergoing 2 h of retrospective optically gated time-lapse acquisition, acquired in a similar manner to that described in ref. ^13^. We used our existing light sheet set-up, but with the laser turned on continuously, to provide illumination equivalent to the acquisition of 600 video frames (each 2.5 ms exposure) per $z$ plane (full details in Supplementary Table 1).

Heart rate was calculated every 5 min using the period-determination codes described in ref. ^11^. Any heart rate determined that varied by more than ±25% from the previous heart rate value was discounted and a new period determined, which always varied by less than this amount. Such events occurred infrequently and were due to changes in heart rhythm causing erroneous period determination; eight of these events occurred, all during the retrospective optical gating protocol.

For each protocol (adaptive prospective optical gating and retrospective optical gating) fish were randomly selected from a full clutch of eggs. All fish were given 30 min to acclimatise, from which a resting heart rate was determined. Fish were then imaged for 2 h and the heart rate measured at the start of each new stack.

For the adaptive prospective optical gating protocol, fish were mounted in the microscope and, after acclimatisation, were exposed to pulsed laser illumination to accompany triggered (synchronised) fluorescence image acquisition. For retrospective gating experiments, fish were mounted in the microscope and, after acclimatisation, were exposed to constant laser illumination as required for retrospective optical gating (see above). In all cases the fish were also exposed to the infrared illumination needed for brightfield imaging (required for heart rate determination as well as for synchronisation analysis).

We note that control fish exposed to no laser light at all show no change in heart rate, similar to the results seen in Fig. 5a for adaptive prospective optical gating experiments. We observed no developmental abnormalities in samples imaged using our method, and fish appear to undergo normal development with no obvious delay for fish developing at 23 °C.

### Assessment of photobleaching during imaging

For retrospective gating, the same method was followed as above. For convenience and consistency, rather than performing an actual retrospective gating analysis on a high-speed recording of fluorescence images, we in fact used our own algorithms to trigger synchronised acquisition of the images that were subsequently analysed and compared against results from adaptive prospective optical gating, but throughout the $z$ scan the laser was left on continuously to provide exactly the same illumination conditions as would be required for retrospective gating acquisition.

To quantify the fluorescence signal level in the $z$ stack, an estimate of the global background signal level was made and subtracted from all pixels. The values of all voxels $v(x,y,t)$ within a volume-of-interest around the heart was summed for each timepoint $t$:

6 $$I(t)=\sum _{xy}v(x,y,t).$$

The resultant time-sequence was fitted with a double-exponential $I(t)=a+b\exp (-ct)+d\exp (-et)$. The constant term $a$ compensates primarily for faint autofluorescence of red blood cells within the heart: due to the constant turnover of blood cells within the light sheet, this signal is not significantly bleached even at extremely high laser doses. We found that the double-exponential provided a good description of the complex dynamics involved in photobleaching, for the time-sequence from the retrospective gating protocol. A single-exponential was fitted in the case of the adaptive prospective gating protocol, since we found that the time constant of any second exponential term was too long to be reliably determined.

### Cardiac laser injury

A Zeiss Photo Activated Laser Microdissection microscope system was used to induce a localised laser injury to the ventricular apex of anaesthetised 72 hpf zebrafish. These were laterally mounted on a glass slide in 20 μl of conditioned water and the laser was fired through a ×20 objective. Individuals were deemed injured when ventricular contractility considerably decreased and the ventricular apex reduced in size without rupturing the myocardial wall. Uninjured (control) fish were treated in the same manner up to the point of laser injury, when they were separated and maintained in the same conditions as injured fish.

### Neutrophil tracking in time-lapse experiments

Neutrophil tracking was performed using the automated algorithms available in FIJI^52^ via the TrackMate plug-in^53^. Detection parameters were: expected diameter 12 μm and quality threshold 20.0. Approximately the same number of neutrophils per frame were detected for all time intervals (Supplementary Fig. 3g). Tracks were then recovered with the Simple Linear Assignment Problem algorithms. At 3 min time-lapse intervals, a maximum linking distance of 30 μm was used, and a gap-closing maximum distance of 60 μm and 2 frames was used. These settings accommodate a maximum neutrophil velocity of 10 μm/min. Detections for all time intervals (see below) and tracks at 3 min intervals were manually inspected to check there were no obvious errors.

To demonstrate the need for time-lapse intervals as short as 3 min, we examined how track features change with increasing time-lapse intervals. First we created two track sets, which we refer to as “subsampled” and “decimated”:

1. Subsampled virtual experiments were performed by starting with 3 min time-lapse dataset but retaining only every second stack for a 6 min interval, every third stack for a 9 min interval, etc. Tracks were recovered for these subsampled experiments as above, except the linking maximum distance and gap-closing maximum distance were scaled to allow the same maximum neutrophil velocity to be captured (Supplementary Fig. 3c shows an example at 30 min intervals). This track set represents our own best attempt at recovering tracks from data taken at longer time intervals, using off-the-shelf analysis tools.
2. Starting from tracks recovered from the 3 min time-lapse dataset, decimated tracks were created by only keeping track points associated with even-numbered stacks for a 6 min interval, and so on. Note that these decimated tracks are the closest to a “ground truth” dataset that can be recovered; it is intuitively apparent that the tracks captured at 3 min will have the fewest accidental track breakages or incorrect linkages (see Supplementary Fig. 3d for an example at 30 min intervals). This track set therefore represents a theoretical upper bound on possible performance (which would likely be impossible to attain in practice), in which the track analysis was actually performed with additional information (3 min interval image stacks) available to ensure correct track linking—that information would not in reality be available in an experiment using longer time-lapse intervals.

From these two track sets we measured the number of neutrophils per frame as a control (Supplementary Fig. 3g), the number of tracks, and the number of tracks that pass through the wound area (Supplementary Fig. 3e, f). We also measured the average absolute velocity and the meandering index^27^ for each individual track (Supplementary Fig. 3h, i).

### Image acquisition, processing and analysis

Volume datasets are presented as maximum intensity projections (MIPs) unless otherwise stated. A trivial linear colour mapping, starting at zero, was used in all cases. For the local detail presented in Figs. 4 and 6, MIPs were computed over a cropped $z$ range to exclude background clutter.

For time series visualisations (apart from Supplementary Video 10), a small exponential correction was applied as a function of time, to compensate for gradual photobleaching. The intensity of all pixels in frame $n$ was multiplied by $\exp (n/\tau )$. $\tau$ was just approximated empirically: for Fig. 6 we used $\tau =250$ for the red (mKate) channel and $\tau =330$ for the green (GFP) channel, for Fig. 3 we used $\tau =500$, and no correction was used in other movies.

Surface rendering was performed using the Imaris software (Bitplane AG). Percentage increase in endothelial chamber volume was calculated by manually cropping the rendered atrium and ventricle and exporting the volume at 48 and 72 hpf.

Image acquisition parameters used for all experiments can be found in Supplementary Table 3.

### Software and data handling

The computer code for real-time synchronisation, adaptive prospective optical gating, and image acquisition is implemented on a Unix-like platform (iMac 2015, Apple Inc.) to ensure consistent real-time performance and to benefit from high-quality performance diagnostic tools. The real-time computational demands of the analysis allow it to be run on a standard desktop computer, and we have even achieved good results using low-end portable computers (iBook 2008, Apple Inc.). The computer code implements the algorithms described above, and presents a graphical user interface for imaging, microscope control and initial post-experiment visualisation and processing (Supplementary Fig. 7). In future we intend to migrate our now-mature synchronisation system to a cross-platform framework such as Micromanager.

Data are streamed to the computer’s hard disk in real-time. The storage requirements are comparatively modest (approx 100 MB/channel/3D timepoint uncompressed) since the only fluorescence images acquired and stored are from the desired phase in the heartbeat. This contrasts with alternative postacquisition approaches^12,13^ that typically require several hundred times this number of images to be recorded for subsequent postprocessing. Maximum intensity projection images of the $z$-stacks are available for monitoring during acquisition, and on experiment completion data are prepared in a format suitable for importing into visualisation software such as Imaris (Bitplane AG).

### Reporting summary

Further information on research design is available in the Nature Research Reporting Summary linked to this article.

## Supplementary information

Supplementary Information

Peer Review File

Description of Additional Supplementary Files

Supplementary Movie 1

Supplementary Movie 2

Supplementary Movie 3

Supplementary Movie 4

Supplementary Movie 5

Supplementary Movie 6

Supplementary Movie 7

Supplementary Movie 8

Supplementary Movie 9

Supplementary Movie 10

Supplementary Movie 11

Reporting Summary

## Data Availability

The data used to produce all results in this paper have been deposited in the University of Glasgow data repository at 10.5525/gla.researchdata.729. This repository contains maximum intensity projections of the fluorescence data shown in: Figs. 3–6; Supplementary Figs. 3 and 5; and Supplementary Videos 4–8 and 10 and 11. Brightfield reference heartbeats used in Figs. 3 and 5, and Supplementary Videos 2 and 9, are also available in this repository. Raw datasets for the experiments in this paper are very large, even using our method, and so most of these are not included in the repository but are available on reasonable request from the corresponding author. However, the full raw data for a small illustrative time range of Fig. 6 have been included in this repository. The repository also includes some processed data for the Jupyter Notebooks provided; this has been described in these Notebooks.

## Code availability

The graphical user interface and all codes required to run adaptive prospective optical gating live have been deposited in the University of Glasgow data repository at 10.5525/gla.researchdata.729. This includes codes for prospective optical gating, updating reference frames and the codes needed for hardware interactions. Also available are Python/Jupyter notebooks for recreating the plots in Figs. 3–5 and Supplementary Figs. 3 and 4, and also for generating Supplementary Video 2.
