# Author Correction: Adaptive prospective optical gating enables day-long 3D time-lapse imaging of the beating embryonic zebrafish heart

**DOI:** 10.1038/s41467-020-17483-z

**Published:** 2020-07-15

**Authors:** Jonathan M. Taylor, Carl J. Nelson, Finnius A. Bruton, Aryan Kaveh, Charlotte Buckley, Carl S. Tucker, Adriano G. Rossi, John J. Mullins, Martin A. Denvir

**Affiliations:** 1 School of Physics and Astronomy, University of Glasgow, Glasgow, UK; 2 British Heart Foundation Centre for Cardiovascular Science, Queen’s Medical Research Institute, University of Edinburgh, Edinburgh, UK; 3 Centre for Inflammation Research, University of Edinburgh Medical School, Teviot Place, Edinburgh, EH8 9AG UK

**Keywords:** Time-lapse imaging, Light-sheet microscopy, Software, Light-sheet microscopy

Correction to: *Nature Communications* 10.1038/s41467-019-13112-6, published online 15 November 2019.

The original version of this Article contained an error in the spelling of the author Aryan Kaveh, which was incorrectly given as Aryan Kaveh Baghbadrani. This has now been corrected in both the PDF and HTML versions of the Article.

