# Supplementary material for: Adaptive prospective optical gating enables day-long 3D time-lapse imaging of the beating embryonic zebrafish heart
Source: Nat Commun. 2019 Nov 15;10:5173. doi: 10.1038/s41467-019-13112-6 (PMC6858381; doi:10.1038/s41467-019-13112-6)
Supplement: Supplementary file 1 — Supplementary Information [file 41467_2019_13112_MOESM1_ESM.pdf]

Adaptive prospective optical gating enables day-long 3D time-lapse imaging of the beating embryonic zebrafish heart

Jonathan M. Taylor*, Carl J. Nelson, Finnius A. Bruton, Aryan K. Baghbadrani, Charlotte Buckley, Carl S. Tucker, Adriano G. Rossi, John J. Mullins, and Martin A. Denvir

*jonathan.taylor@glasgow.ac.uk

October 2, 2019

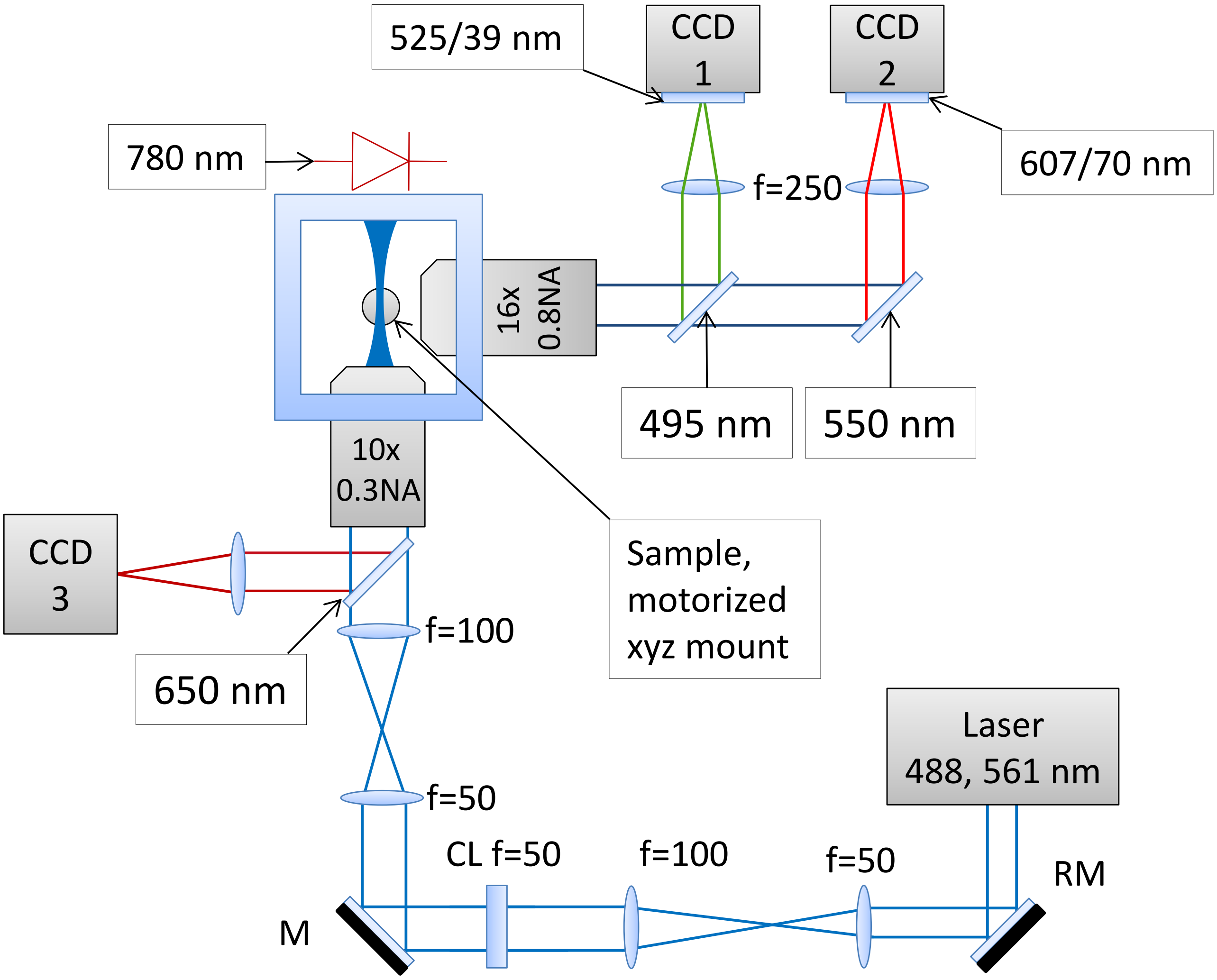

Supplementary Figure 1: **Optical setup for microscope used to demonstrate adaptive prospective optical gating.** A light sheet is launched into the sample chamber via a water-dipping objective lens. The same objective lens is simultaneously used for brightfield near-infrared imaging (CCD3), illuminated by the 780nm LED and picked off by the 650nm dichroic mirror. A separate water-dipping objective lens is used for imaging fluorescence in multiple colour channels (CCD1, 2). The adaptive prospective optical gating algorithms analyze the brightfield images from CCD3, and generate trigger signals that cause synchronized acquisition of individual fluorescence frames by CCD1 and CCD2. Lasers are pulsed such that they only illuminate the sample during the exposure window of the relevant CCD camera. RM: resonant galvanometer mirror implementing mSPIM technique to minimize shadows through angular diversity of light sheet propagation. CL: cylindrical lens forming the light sheet. M: mirror used to position light sheet parfocal to imaging objective.

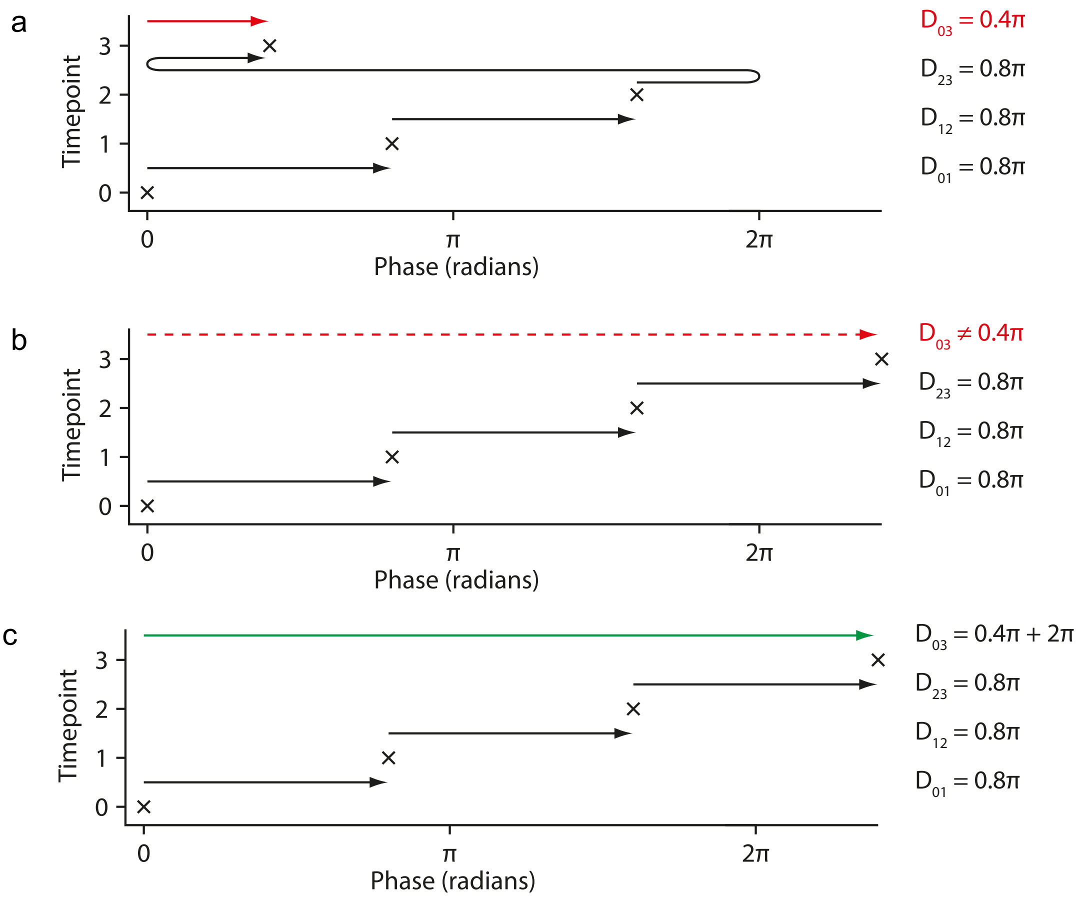

Supplementary Figure 2: **Phase-wrapping impedes global sequence alignment. a–b,** When considering more than just nearest-neighbour relative shifts (a), the presence of phase-wrapping will result in contradictions (b) that do not permit a consistent global shift solution to be computed. Here, the relative shift between sequences 0 and 3 cannot be reconciled with the relative shifts $D_{01}+D_{12}+D_{23}$. No choice of sign convention etc can eliminate this contradiction in all circumstances. **c,** Our multi-pass sequence alignment algorithm first considers adjacent relative shifts, and then adjusts longer-range relative shifts to be consistent with them. Inclusion of (adjusted) longer-range shifts is crucial to avoid accumulated random errors over the course of a time-lapse sequence.

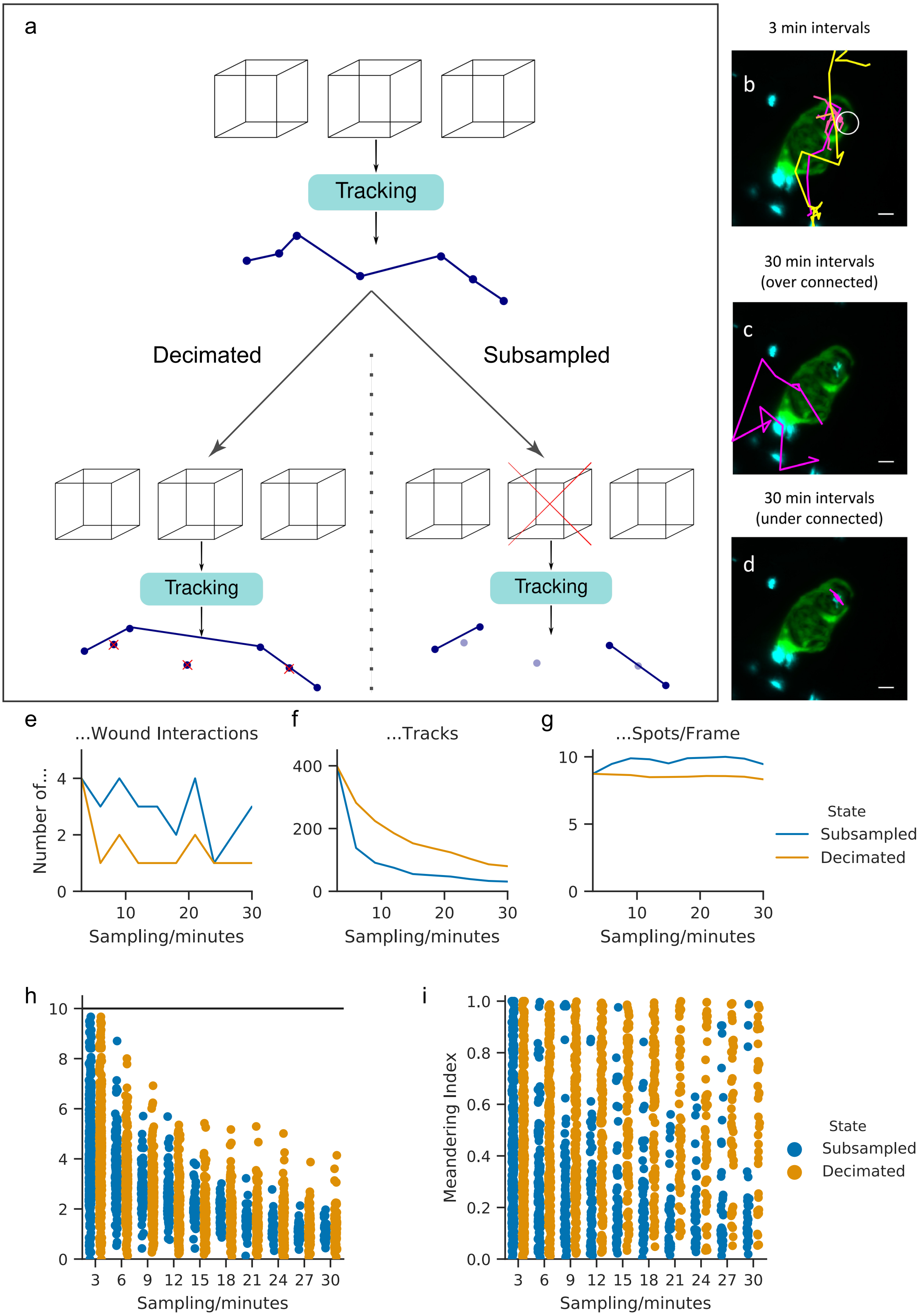

Supplementary Figure 3: **Short-interval 3D time-lapse imaging, only possible with adaptive prospective optical gating, is essential for accurate cell tracking.** [full caption overleaf...]

Supplementary Figure 3: **Short-interval 3D time-lapse imaging, only possible with adaptive prospective optical gating, is essential for accurate cell tracking. a,** We tested the effect of different sampling intervals by starting with a 'ground truth' dataset sampled at 3 minute intervals, and generating downsampled tracks in two different ways. We *decimated* tracks recovered from the 3 minute dataset, to produce tracks with lower temporal resolution but with correct temporal linkages. We also generated tracks directly from analysis of *sub-sampled* time-lapse data; these sub-sampled tracks were recovered in the same way as ground truth tracks, but the LAP algorithm was allowed a proportionately larger search radius. **b,** MIP demonstrating that the high temporal acquisition rate permits track analysis (coloured lines) of rapidly migrating neutrophils (cyan - transgene *mpx*:mCherry) at the injury site (white circle) on the apex of the ventricle (green - transgene *myl7*:GFP). At 3 minute intervals, a rate only feasible with our adaptive prospective optical gating, we can clearly track two neutrophils that interact at the wound site (and two more that pass through without interacting). Image is a still frame from Supplementary Video 7. Injury was induced at 72 hpf, with imaging commencing ∼74 hpf. **c–d,** However, with larger time intervals, here 30 minutes as commonly used in other longitudinal cardiac imaging studies, tracking algorithms yield very short incorrectly connected tracks (d) due to the now large frame-to-frame movement of cells, or erroneous and erratic paths (c) due to the large distances travelled by cells between each timepoint. In both scenarios (c and d) the captured tracks miss important details of the neutrophil migratory behaviour. **e–f,** Longer sampling intervals dramatically degrade the accuracy of track recovery (f) and result in an incorrectly low number of detected wound region interactions (e), due to tracking failures with fast neutrophils, and missing of transient events. **g,** Number of detections per frame when comparing subsampled image data (blue) against 'ground truth' decimated tracks (orange). This plot serves as a control confirming that the decimated and subsampled data are using similar detected nuclei. **h-i,** Tracking errors at longer sampling intervals cause quantification errors when trying to assess neutrophil behaviour. The average absolute velocity (h) and meandering index (i) are inherent properties of the neutrophils observed, and the dramatic change in distribution for larger sampling intervals represents incorrect quantification, emphasizing that short sampling intervals are essential for correct results. For the velocity, the fastest neutrophils are lost and the average velocity across the tracks drops; in the subsampled case, erroneous track recovery also loses tracks with very low velocity as these are incorrectly merged with other tracks. The meandering index (a measure of track tortuosity, a common proxy for cellular interactions [1]) is also incorrectly computed. Scale bars: 30μm.

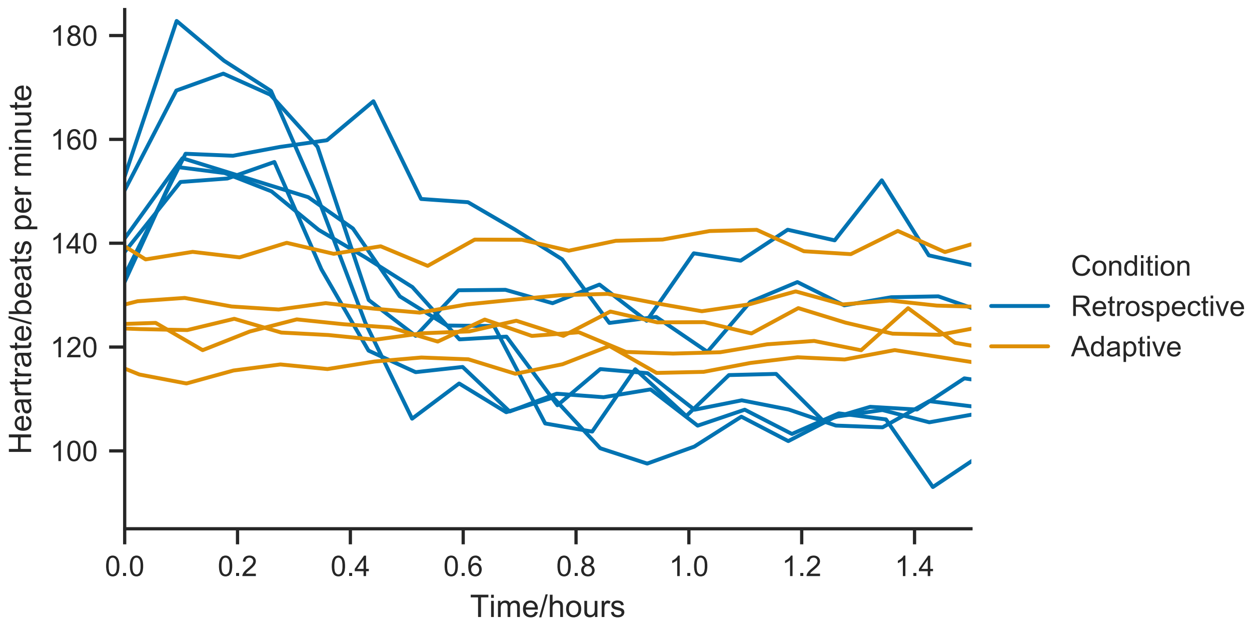

Supplementary Figure 4: **Adaptive prospective optical gating protocol causes less cardiac photo-injury and photo-bleaching than retrospective gating protocols.** A replotting of Figure 5a showing individual fish. When multiple fluorescent z-stacks in an embryonic heart (transgene *myl7*:GFP; 3 dpf) using a retrospective optical gating protocol (blue lines; $n = 6$ fish), changes in heart rate are seen (discussed in main text). Meanwhile image acquisition using our adaptive prospective optical gating protocol (orange; $n = 5$ fish) with stacks acquired every 5 min, causes no notable change in heart rate.

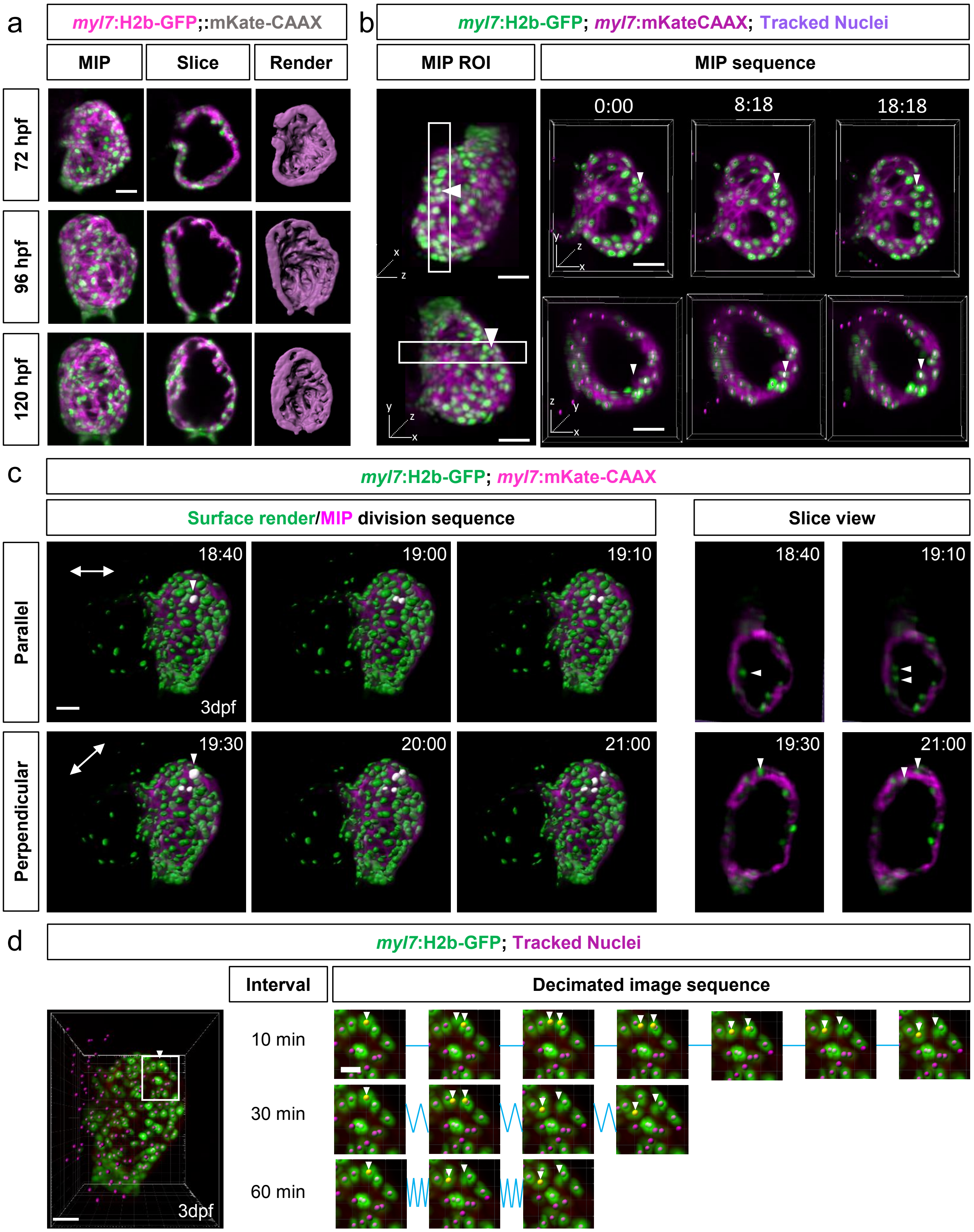

Supplementary Figure 5: **Adaptive prospective optical-gating yields correct interpretations of trabecular development at the tissue and cellular level.** [full caption overleaf...]

Supplementary Figure 5: **Adaptive prospective optical-gating yields correct interpretations of trabecular development at the tissue and cellular level.** **a,** A single *myl*:h2b-GFP;*myl7*:mKate-CAAX ventricle imaged in three discrete 6 hour time-lapses beginning at 3dpf, 4 dpf and 5dpf. Representative images from each time-lapse showing the developing trabecular network. Cardiomyocyte nuclei are shown in magenta and cardiomyocyte membranes in gray. **b,** Some trabeculation events were also confirmed to take place without division, by delamination alone. When examined in orthogonal planes, a single cardiomyocyte nuclei (white arrowhead) can be tracked moving luminally out of the compact myocardial layer. Magenta dots represent detected and tracked nuclei. **c,** Comprehensive investigation of 3D time-lapse datasets reveal two categories of mitotic events, where division takes place parallel (top) or approximately perpendicular (bottom) to the myocardial wall (dividing cells highlighted in white). Only by examining the time series in oblique 3D slice views (in the plane of division) can we correctly identify the nature of the division. Representative timepoints from a time-lapse of whole 3dpf heart, showing surface-rendered cardiomyocyte nuclei (green - transgene *myl7*:h2b-GFP) superimposed onto a MIP of cardiomyocyte membranes (magenta - transgene *myl7*:mKate-CAAX). **d,** With longer-interval time-lapses, the true origins of recently-divided cells are soon obfuscated as they pass by neighbouring cells. Scale bars: 30μm.

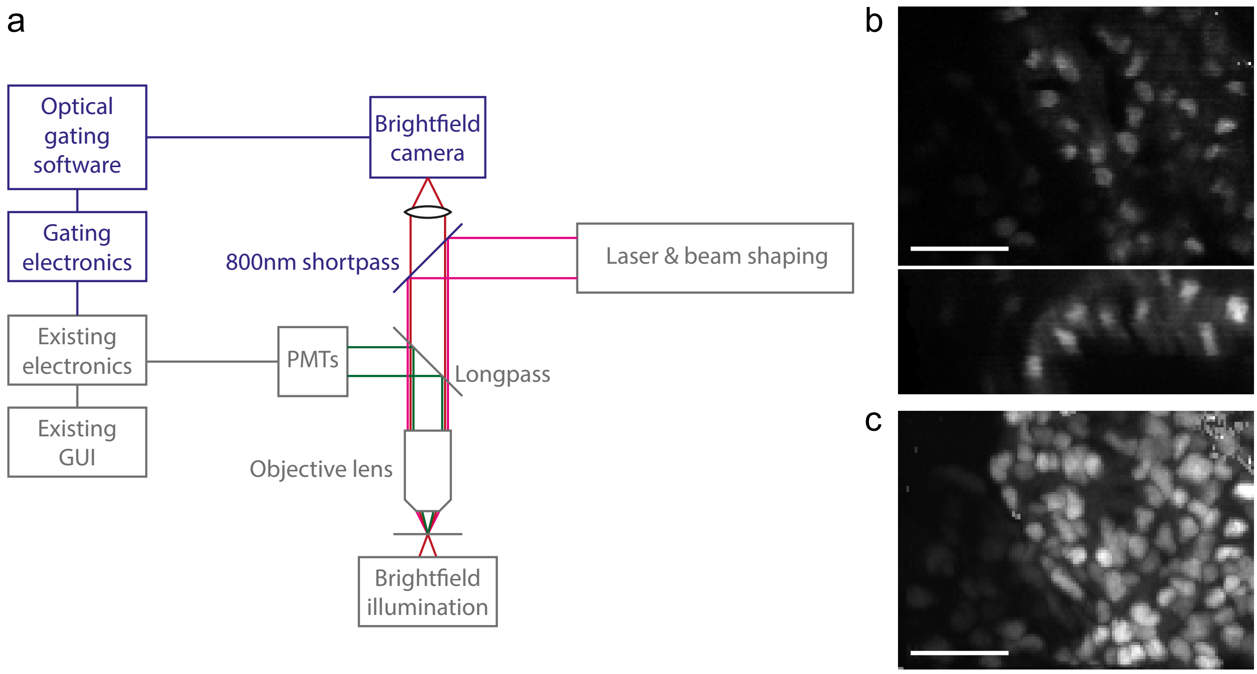

Supplementary Figure 6: **Synchronized two-photon microscopy. a,** Schematic diagram of Scientifica commercial two-photon microscope (grey) and the minor modifications (black) required to integrate our prospective optical gating system. **b–c,** xy and xz projections (b) and maximum intensity projection (c) of synchronized two-photon z stack, showing cardiomyocyte nuclei (transgene *myl7*:h2b-gfp) in region of interest in the zebrafish ventricle (5 dpf). Scale bars: 30μm.

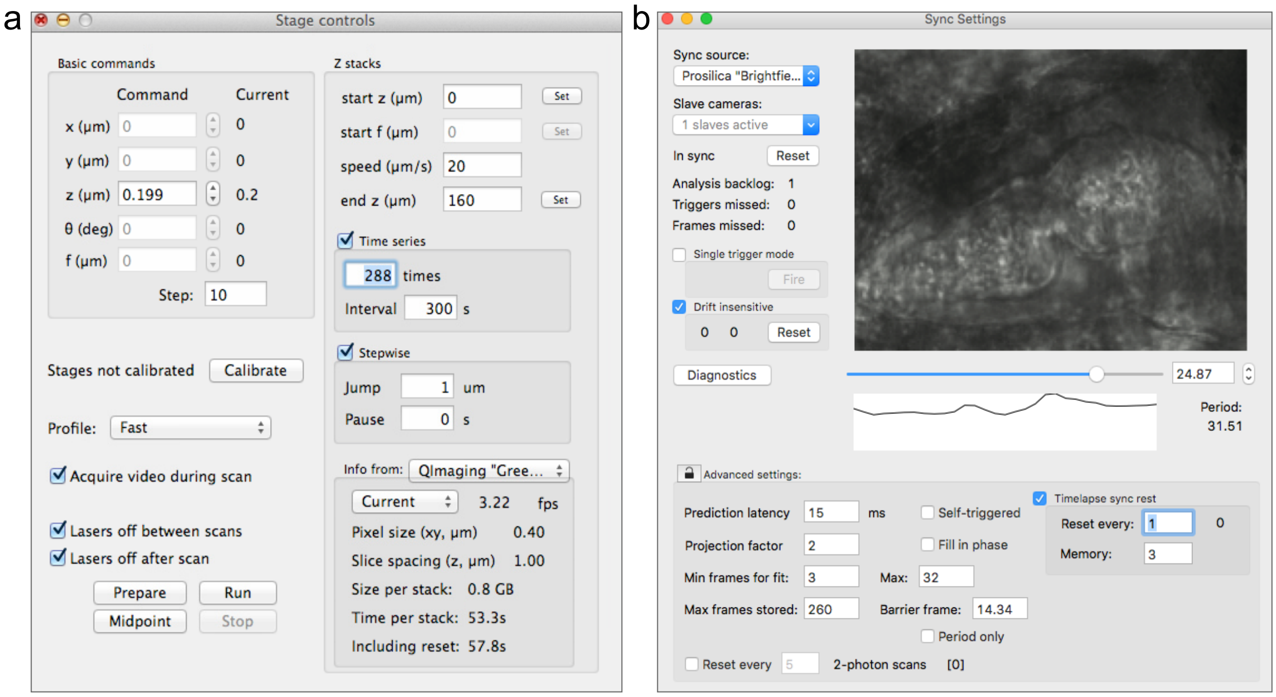

Supplementary Figure 7: **User interface for adaptive prospective optical gating.** Example screenshots from our complete graphical user interface for time-lapse optically-gated imaging experiments. **a,** Time-lapse z stacks are programmed to repeat at regular intervals, with uniformly-spaced z slices acquired at a fixed phase in the cardiac cycle. **b,** Optical gating is configured by the user at the start of the experiment, and it then runs without further user intervention. Key features include: selection of 'slave' cameras for triggered acquisition; selection of target phase in the cardiac cycle; regular updates of the image sequence representing our reference heartbeat ("time-lapse sync reset") in order to adapt to the changing appearance of the heart over developmental timescales.

Table 1: The following table contains the key parameters used in the experiments for Figure 5a. Definitions match those given in [2].

Parameter	Units	Adaptive Prospective Optical Gating	Retrospective Optical Gating[1]
Wavelength	nm	488	
Illumination Time (per individual frame)	ms	2.5	
Dark Interval (between frames)	ms	400[2]	0[3]
Stack Time	s	82[2]	225
Dark Interval (between stacks)	s	200[2]	75
Number of Images per Plane		1	600
Number of Planes per Stack[4]		150	
Number of Stacks		20	
Distance between z planes	μm	1	
Excitation power[5]	mW	1.25	
Intensity[5]	Wcm^{-2}	118	
Exposure	Jcm^{-2}	0.3	450
Energy per stack	J	0.00019	0.28

[1] Parameters selected to give a light dose similar to that used in [3].

[2] The minimum time between individual slice captures is one heartbeat (approximately 140 beats per minute) but this interval will vary with age and health and photoresponse. This will also affect the total time to collect a whole stack.

[3] The retrospective optical gating protocol uses constant laser illumination for back-to-back image capture.

[4] The size of images and number of images per stack were varied depending on the orientation and size of individual hearts.

[5] We measured our light sheet to be approximately 2.4 μm thick (FWHM) and 390 μm tall (FWHM) at its centre. We measured that approximately 11% of the power configured in the laser software arrived at the sample, after optical losses.

Table 2: The following table contains the key parameters used in the experiments for Figure 5b and Supplementary Video 9. Definitions match those given in [2].

Parameter	Units	Adaptive Prospective Optical Gating	Retrospective Optical Gating[1]
Wavelength	nm	561	
Illumination Time (per individual frame)	ms	15	
Dark Interval (between frames)	ms	400[2]	0[3]
Stack Time	s	60[2]	240
Dark Interval (between stacks)	s	240[2]	60
Number of Images per Plane		1	106
Number of Planes per Stack[4]		140	150
Number of Stacks		262	29
Distance between z planes	μm	1	
Excitation power[5]	mW	1.2	
Intensity[5]	Wcm^{-2}	113	
Exposure	Jcm^{-2}	4.3	460
Energy per stack	J	0.0025	0.29

[1] Parameters selected to give a light dose similar to that used in [3].

[2] The minimum time between individual slice captures is one heartbeat (approximately 140 beats per minute) but this interval will vary with age and health and photoresponse. This will also affect the total time to collect a whole stack.

[3] The retrospective optical gating protocol uses constant laser illumination for back-to-back image capture.

[4] The size of images and number of images per stack were varied depending on the orientation and size of individual hearts. For this reason, the number of planes is slightly different between the two fish here.

[5] We measured our light sheet to be approximately 2.4 μm thick (FWHM) and 390 μm tall (FWHM) at its centre. We measured that approximately 11% of the power configured in the laser software arrived at the sample, after optical losses.

Table 3: The following table contains the key parameters used in the experiments for all figures. Definitions match those given in [2].

Parameter	Units	Figure			
		3a (Supp. Video 4)	4a (Supp. Video 5)	4b (Supp. Figure 4, Video 6)	6 (Supp. Figure 5, Video 7)
Fluorophore: Wavelength	nm	mCherry:561	GFP:488; mCherry:561	GFP:488; mCherry:561	GFP:488; mKate:561
Illumination Time (per individual frame)	ms	30	7; 15	7; 30	10; 15
Dark Interval/ms (between frames)[1]	ms	470	360	470	400
Stack Time/s[1]	s	110	50	32	60
Dark Interval/s (between stacks)[1]	s	190	130	148	240
Number of Images per Plane		1	1	1	1
Number of Planes per Stack		220	131	64	140
Number of Stacks		288	200	460	262
Distance between z planes	μm	1	0.75	1.5	1
Excitation power[2]	mW	11	11	11	11
Intensity[2]	$\mathrm{Wcm^{-2}}$	1010	1010	1010	1010
Exposure	$\mathrm{Jcm^{-2}}$	79	19; 40	19; 79	26; 40
Energy per stack	J	0.073	0.010; 0.022	0.010; 0.043	0.015; 0.023

[1] Numbers are approximate. The minimum time between individual slice captures is one heartbeat (approximately 140 beats per minute; 155 beats per minute for figure 4a) but this interval will vary with age and health. This will also affect the total time to collect a whole stack.

[2] We measured our light sheet to be approximately 2.4 μm thick (FWHM) and 390 μm tall (FWHM) at its centre. We measured that approximately 11% of the power configured in the laser software arrived at the sample, after optical losses.

Supplementary Note 1: brightfield source images and synchronization performance

Any image-based heart synchronization strategy performs best when the appearance of the heart is invariant over time (aside from periodic changes over the heartbeat). It is also desirable to maximize the useful “signal” (features that repeat from one cycle to the next) compared to noise (features such as blood cells that do not repeat from one cycle to the next, as well as true noise such as camera shot noise) and background (features that do not move with the heartbeat).

In synchronized 3D imaging the requirement for invariance gives rise to a conflict between the need to scan the fluorescence image plane through the sample (or vice-versa) for 3D imaging, and the desire to image a fixed plane in order to obtain a consistent phase reference for synchronization. Various strategies for dealing with this issue can be found in the literature:

- Attempting to maintain phase-lock despite gradual changes in appearance from one slice to the next [4] (but this approach suffers from accumulated systematic error in z [3, 5])
- Refocusing the brightfield images using a motorized mobile tube lens [6] (but this is cumbersome and in practice limits acquisition to a z range of 120 μm).
- Use of a rotational imaging strategy, with synchronization being based on a line of pixels along the axis of rotation [5].

In the present work we instead acquire our phase-reference images via brightfield imaging through the light sheet launch objective. Consequently, during a z scan the brightfield images remain focused on the same plane in the sample, but the image translates within the focal plane of the brightfield camera. Our heart synchronisation algorithms are already tolerant of such in-plane motion (to accommodate possible sample drift), and thus we are able to maintain a high-quality phase-lock throughout a whole z scan.

Because z scanning leads to translation within the focal plane of the brightfield camera, we need to use a region of interest on the (static) camera sensor that is larger than just the size of the heart itself. To avoid this increasing the size of the brightfield images that must actually be analyzed by our algorithms, we implement an adaptive region of interest in software. The region of interest actually read out from the camera is larger than the heart, but we then discard pixels from outside a moving region of interest (whose location is computed based on knowledge of the current z coordinate of the sample translation stage). The result is that the images our algorithms actually process are not any larger than they would need to be for the case of a non-translating sample.

Here we have not specifically investigated strategies for maximizing “signal” levels, although other authors have noted the fact that wavelet filtering may eliminate some non-periodic contributions to the signal [4], and we have found darkfield imaging useful for ultra-compressed (7 pixel) measurements [7]. Other imaging modalities such as phase contrast may also merit consideration, although the simplicity of implementation of brightfield imaging makes it attractive as a solution. Averaging phase-reference images over multiple heartbeats might be beneficial in reducing “noise” levels due to the appearance of blood cells, although our preliminary investigation [unpublished] suggests that the presence of blood cells is not a limiting factor on synchronization performance.

With our current algorithms, the quality of phase-lock does tend to degrade towards the end of a 24h acquisition. We have found that factors include:

- In many cases, anaesthesia begins to wear off (not being maintained at a stable level over 24h) leading to small movements of the embryo in its mounting agarose, which cause “glitches” with the synchronization. Further research is required to optimize anaesthesia and life support protocols over these timescales.
- Gradual movement of specimen, due to small motion of whole embryo in its mounting agarose, or due to growth of whole embryo causing heart to move slightly from its initial position. We have discussed how our algorithms are insensitive to drift, but movement can still become a problem eventually, if the heart is not fully inside field of view, or is no longer in sharp focus.
- In the case of laser-ablation-injured fish, increases in (slight) arrhythmia as a consequence of injury and subsequent inflammatory response will reduce the precision of our current prospective gating algorithms (as noted in the caption for Supplementary Video 6)
- At around 2-3dpf, temporary degradation of image quality (brightfield as well as fluorescence) occurs due to development of what we believe to be “hatching gland” structures [8, Figure 32].

Indeed, without our described strategy for accommodating sample drift, these first two items would be showstoppers. We expect to increase our understanding of the relative importance of these factors as we acquire more and more datasets and are able to study the performance of the algorithms over even larger numbers of experiments.

References

[1] Katherine M Henry et al. "PhagoSight: an open-source MATLAB® package for the analysis of fluorescent neutrophil and macrophage migration in a zebrafish model", *PloS one* **8** (2013), e72636.

[2] P Philippe Laissue et al. "Assessing phototoxicity in live fluorescence imaging", *Nature Methods* **14** (2017), pp. 657–661. DOI: 10.1038/nmeth.4344.

[3] Michaela Mickoleit et al. "High-resolution reconstruction of the beating zebrafish heart", *Nature Methods* **11** (2014), pp. 1–6. DOI: 10.1038/nmeth.3037.

[4] Michael Liebling et al. "Four-dimensional cardiac imaging in living embryos via postacquisition synchronization of nongated slice sequences", *Journal of Biomedical Optics* **10** (2005), p. 054001. DOI: 10.1117/1.2061567.

[5] Irina V Larina et al. "Sequential Turning Acquisition and Reconstruction (STAR) method for four-dimensional imaging of cyclically moving structures", *Biomedical Optics Express* **3** (2012), pp. 650–60. DOI: 10.1364/BOE.3.000650.

[6] Jonathan M Taylor, John M Girkin, and Gordon D Love. "High-resolution 3D optical microscopy inside the beating zebrafish heart using prospective optical gating", *Biomedical Optics Express* **3** (2012), pp. 3043–53. DOI: 10.1364/BOE.3.003043.

[7] J.M. Taylor, A.L. Hargreaves, and S. Daryanavard. "Microscopy without imaging: Compressive sensing for heart-synchronized imaging", *OSA Technical Digest*. 2017, CTh3B.2. ISBN: 9781557528209. DOI: 10.1364/COSI.2017.CTh3B.2.

[8] Charles B Kimmel et al. "Stages of Embryonic Development of the Zebrafish", *Developmental Dynamics* **203** (1995), pp. 253–310.

Supplementary Note 2: hardware interfacing

Timing hardware

Typical desktop computers do not have a standardised interface for simple digital input/output, suitable for communicating with hardwares such as cameras. Furthermore, it is not trivial to write code with guaranteed predictability to enable millisecond-resolution accuracy in hardware interactions. For these reasons, our platform is design to interface over an RS-232 serial link to a custom-built microcontroller system that generates the actual electrical trigger signals at the times determined by our synchronization software. In practice, this timing hardware fulfils other roles as well, pulsing lasers in time with camera exposures, generating custom digital waveforms for particle image velocimetry, etc. A simple text-based communications protocol is defined (see document "communications protocol.rtf" in supplementary source code) for communication between the main computer software and the timing hardware.

Source code and schematics for our custom timing hardware have been made available, but any hardware conforming to the specified communications protocol could be used. Other hardware with compatible capabilities could be used by adapting our source code (new subclass of TimingBoxBase, modelling code on existing subclasses TimingBoxXMOS and TimingBoxFPGA) to support a different communications protocol.

Camera support

Currently our user interface supports the following camera models:

- Prosilica (recommended as brightfield source for heart synchronization) via PvAPI driver for GigE cameras
- Ximea (XiD, XiQ) via xiAPI
- QImaging via deprecated QCam API

Other camera hardware and drivers can be supported by subclassing CameraBase/FrameBase classes, modelling code on the existing camera support classes.

Translation stage support

Currently our user interface supports the following translation stage models:

- Physik Instrumente via the Mercury native command interface
- Newport via SMC100 command set

Other stage hardware can be supported by subclassing the StageCommandInterface class, modelling code on existing stage support classes.
