# Supplementary material for: Adaptive prospective optical gating enables day-long 3D time-lapse imaging of the beating embryonic zebrafish heart
Source: Nat Commun. 2019 Nov 15;10:5173. doi: 10.1038/s41467-019-13112-6 (PMC6858381; doi:10.1038/s41467-019-13112-6)
Supplement: Supplementary file 3 — Description of Additional Supplementary Files [file 41467_2019_13112_MOESM3_ESM.pdf]

Description of Additional Supplementary Files

Supplementary Movie 1: **Optical gating concept.** The constant motion of th
ebeating heart presents a challenge for imaging (grey representation of part of t
heheart ventricle). Light sheet fluorescence microscopy provides optical section
ingto image a single plane of the heart at a time (depicted in green), with
outany superfluous illumination in other spatial locations. However, retrospec
tiveoptical gating techniques require many raw fluorescence video images t
o beacquired at *every* z plane in the heart; a consistent z stack at a fixed ca
rdiacphase is reconstructed following subsequent computer analysis. In con
trast,prospective optical gating ensures that the heart is only exposed to poten
tially-harmful laser light at the required *time* in the cardiac cycle.

Supplementary Movie 2: **Prospective optical gating fails to maintai
nphase-lock over extend periods.** Due to changes in brightfield feature
s,prospective optical gating loses phase-lock with time (left) whilst adapti
veprospective optical gating is able to maintain a lock (right) over many ho
urs.The colours indicate the difference in phase between the frame shown and
themean human-scored target frame (c.f. Figure 3). Scale bar: 30μm.

Supplementary Movie 3: **Diagrammatic representation of adaptiv
eprospective optical gating.** For each time-lapse point, a new reference hear
t-beat is determined in the brightfield images. This new reference heartbeat cou
ldbe used to create a 3D stack using prospective optical gating; however, the n
ewreference heartbeat is not aligned with previous reference sequences (blue).
Byincorporating retrospective optical gating on these brightfield reference he
artbeats, including motion correction and a weighted regression with historical
ref-erence sequences, the previous target can be identified in each new refer
encesequence. This allows phase-locking between stacks throughout a time-
lapseexperiment (right).

Supplementary Movie 4: **Phase-locked, day-long cardiac time-lapse imag
-ing of cardiac morphogenesis.** Phase-locked, live time lapse imaging of hea
rtdevelopment (48 to 72 hours post-fertilization (hpf), at 300s intervals) showi
ngendothelium lining blood vessels and heart chambers (transgene *flk1:mCherr
y*).Movie shows maximum intensity projections of z-stacks. Scale bar: 30μm.

Supplementary Movie 5: **Phase-locked, day-long cardiac time-lapse imag
-ing of cardiac morphogenesis.** Phase-locked, live time lapse imaging of hea
rtdevelopment (48 to 72 hours post-fertilization (hpf), at 300s intervals) showi
ngendothelium lining blood vessels and heart chambers (transgene *flk1:mCherr
y*).Movie shows 3D rendering of heart chambers. Scale bar: 30μm.

Supplementary Movie 6: **Sustained beating-heart time-lapse imaging o fimmune cell responses to cardiac injury.** Synchronized time-lapse 3 Dimaging of macrophage immune cells migrating to the site of laser-injury in azebrafish heart showing intensive macrophage activity at the wound site. Ma x-imum intensity projection of *z*-stacks, showing macrophages (magenta - tra ns-gene *mpeg:mCherry*) at the injury site on cardiomyocytes (green - transg ene*myl7:GFP*). Injury occurred at 72 hpf, with imaging commencing one hour af-ter injury; note that the effect of laser injury on heart rhythm posed challen gesfor the synchronization, resulting in some planes and/or timepoints with p oorsynchronization. Scale bar: 30μm, Timestamps: hours post-injury.

Supplementary Movie 7: **Sustained beating-heart time-lapse imaging o fimmune cell responses to cardiac injury.** Synchronized time-lapse 3 Dimaging of macrophage immune cells migrating to the site of laser-injury in azebrafish heart, showing macrophage activity and shape changes at the wou ndsite. Maximum intensity projection of *z*-stacks, showing macrophages (mage nta- transgene *mpeg:mCherry*) at the injury site on cardiomyocytes (green - tr ans-gene *myl7:GFP*). Injury occurred at 72 hpf, with imaging commencing one hourafter injury; note that the effect of laser injury on heart rhythm posed chall engesfor the synchronization, resulting in some planes and/or timepoints with poorsynchronization. Scale bar: 30μm, Timestamps: hours post-injury.

Supplementary Movie 8: **Sustained beating-heart time-lapse imaging o fimmune cell responses to cardiac injury.** Synchronized time-lapse 3 Dimaging of neutrophil immune cells migrating to the site of laser-injury in azebrafish heart. Maximum intensity projection of *z*-stacks, showing neutrophi ls(cyan - transgene *mpx:mCherry*) at the injury site on the apex of the ventric le(green - transgene *myl7:GFP*). Injury was induced at 72 hpf, with imagi ngcommencing two hours after injury. Scale bar: 30μm, Timestamps: hours p ost-injury.

Supplementary Movie 9: **Retrospective gating affects heart function.** W ehave observed changes in heart function when using retrospective gating prot o-cols to acquire time-lapse datasets. Video shows visible deterioration of ejecti onfraction, heart rate and rhythm.

Supplementary Movie 10: **Adaptive prospective optical gating substan -tially reduces bleaching rate in sensitive samples.** When fluorophores ar eparticularly light-sensitive, or labelling is highly specific and therefore spars e(e.g. *myl7:mKate-CAAX* transgenic line), we observe that specimens are easil ybleached, even using light sheet fluorescence microscopy. When exposed to lig htlevels required for retrospective gating (using typical parameters reported in t heliterature), the signal from a specimen labelled with membrane-specific mK atefluorophore is halved after only 3 timepoints, whereas with adaptive prospec tiveoptical gating over 150 timepoints are recorded before the signal level drop s bythe same amount.

Supplementary Movie 11: **3D time-lapse imaging to track cardiomyocyt eproliferation during development.** 24 hour time-lapse (shown here as aMaximum Intensity Projection) of a 72–96hpf embryo, showing cardiomyocy tenuclei (green - transgene *myl7:h2b-gfp*) and cardiomyocyte membranes (magen ta- transgene *myl7:mKate-CAAX*). Nuclei of cardiomyocytes which are about todivide are marked by white arrowheads (open arrowheads for less obvious c elldivisions in the posterior wall of the heart). Scale bar: 30μm.
