# Supplementary material for: Adaptive prospective optical gating enables day-long 3D time-lapse imaging of the beating embryonic zebrafish heart
Source: Nat Commun. 2019 Nov 15;10:5173. doi: 10.1038/s41467-019-13112-6 (PMC6858381; doi:10.1038/s41467-019-13112-6)
Supplement: Supplementary file 15 — Reporting Summary [file 41467_2019_13112_MOESM15_ESM.pdf]

nature research

Corresponding author(s): Jonathan Taylor (Glasgow University), Martin Denvir (Edinburgh University)

Last updated by author(s): Sep 16, 2019

Reporting Summary

Nature Research wishes to improve the reproducibility of the work that we publish. This form provides structure for consistency and transparency in reporting. For further information on Nature Research policies, see Authors & Referees and the Editorial Policy Checklist .

Statistics

For all statistical analyses, confirm that the following items are present in the figure legend, table legend, main text, or Methods section.

n/a	Confirmed	
☐	☒	The exact sample size (n) for each experimental group/condition, given as a discrete number and unit of measurement
☐	☒	A statement on whether measurements were taken from distinct samples or whether the same sample was measured repeatedly
☒	☐	The statistical test(s) used AND whether they are one- or two-sided<br>*Only common tests should be described solely by name; describe more complex techniques in the Methods section.*
☒	☐	A description of all covariates tested
☒	☐	A description of any assumptions or corrections, such as tests of normality and adjustment for multiple comparisons
☐	☒	A full description of the statistical parameters including central tendency (e.g. means) or other basic estimates (e.g. regression coefficient) AND variation (e.g. standard deviation) or associated estimates of uncertainty (e.g. confidence intervals)
☒	☐	For null hypothesis testing, the test statistic (e.g. F, t, r) with confidence intervals, effect sizes, degrees of freedom and P value noted<br>*Give P values as exact values whenever suitable.*
☒	☐	For Bayesian analysis, information on the choice of priors and Markov chain Monte Carlo settings
☒	☐	For hierarchical and complex designs, identification of the appropriate level for tests and full reporting of outcomes
☒	☐	Estimates of effect sizes (e.g. Cohen's d, Pearson's r), indicating how they were calculated

Our web collection on statistics for biologists contains articles on many of the points above.

Software and code

Policy information about availability of computer code

Data collection	Data collection: All data was collected using custom software. This software has been made available with the paper at http://dx.doi.org/10.5525/gla.researchdata.729 (see Online Methods - Code availability).
Data analysis	All analysis codes used to create figures in this paper have been made available as Python/Jupyter notebooks at http://dx.doi.org/10.5525/gla.researchdata.729 (see Online Methods - Code availability). Additionally, we used Imaris (Bitplane AG) to carry out surface rendering and cardiac volume calculations (Figure 3 & 6, and Supplementary Video 5)

For manuscripts utilizing custom algorithms or software that are central to the research but not yet described in published literature, software must be made available to editors/reviewers. We strongly encourage code deposition in a community repository (e.g. GitHub). See the Nature Research guidelines for submitting code & software for further information.

Data

Policy information about availability of data

All manuscripts must include a data availability statement. This statement should provide the following information, where applicable:

- Accession codes, unique identifiers, or web links for publicly available datasets
- A list of figures that have associated raw data
- A description of any restrictions on data availability

The data used to produce all results in this paper have been deposited in the University of Glasgow data repository at http://dx.doi.org/10.5525/gla.researchdata.729. This repository contains maximum intensity projections of the fluorescence data shown in: Figures 3--6; Supplementary Figures 2 and 4; and Supplementary Videos 4--7 and 9--10. Brightfield reference heartbeats used in Figures 3 and 5, and Supplementary Videos 2 and 8, are also available in this repository. Raw datasets for the experiments in this paper are very large, even with hybrid optical gating, and so most of these are not included in the repository but are available on reasonable request from the corresponding author. However, the full raw data for a small illustrative time range from Figure 6 have been included in this repository. The repository also includes some processed data for the Jupyter Notebooks provided; this has been described in these Notebooks.

Field-specific reporting

Please select the one below that is the best fit for your research. If you are not sure, read the appropriate sections before making your selection.

☒ Life sciences ☐ Behavioural & social sciences ☐ Ecological, evolutionary & environmental sciences

For a reference copy of the document with all sections, see nature.com/documents/nr-reporting-summary-flat.pdf

Life sciences study design

All studies must disclose on these points even when the disclosure is negative.

Sample size	The novelty of the approach and experiments described here meant that pre-hoc power calculations were not possible. We found that trends in heartrate were clear with n=5/6, which also agrees with previous work on the effects of drugs and temperature on heart rate [Denvir et al, our ref 17]
Data exclusions	As described in manuscript, data exclusion principles were pre-determined for the heartrate response data in Figure 5a - if period determination failed at any time point it was repeated 0.5 seconds later; this was carried out a total of 7 times across 240 attempts. All other data was included.
Replication	Heartrate response data (Figure 5a) was replicated on two different light sheet microscopes (data not reported) - a change in heart rate was observed for retrospective optical gating and no change was seen for hybrid optical gating on both systems. This was also consistent with other experiments using hybrid optical gating (but not focused on heartrate). Other experiments capturing heart looping, neutrophil and macrophage response and trabeculation are part of larger studies into zebrafish heart biology and the data shown is representative of multiple replicants.
Randomization	For each protocol or experiment, fish were randomly selected from a full clutch of eggs (avoiding individuals that were obviously abnormal on initial visual inspection).
Blinding	Automatic analysis codes, including exclusion principles, for extracting heartrate information were written and tested before carrying out the experiment in Figure 5a. Data from these fish could then be analysed without any human intervention thus removing the need for investigator blinding for this analysis.

Reporting for specific materials, systems and methods

We require information from authors about some types of materials, experimental systems and methods used in many studies. Here, indicate whether each material, system or method listed is relevant to your study. If you are not sure if a list item applies to your research, read the appropriate section before selecting a response.

Materials & experimental systems

n/a	Involved in the study
☒	☐ Antibodies
☒	☐ Eukaryotic cell lines
☒	☐ Palaeontology
☐	☒ Animals and other organisms
☒	☐ Human research participants
☒	☐ Clinical data

Methods

n/a	Involved in the study
☒	☐ ChIP-seq
☒	☐ Flow cytometry
☒	☐ MRI-based neuroimaging

Animals and other organisms

Policy information about studies involving animals; ARRIVE guidelines recommended for reporting animal research

Laboratory animals	The study involved embryonic zebrafish (Danio rerio) [sex indeterminable due to age]. A range of transgenic lines were used (specified in manuscript), ages 2-5 days post fertilization. Details stated in manuscript.
Wild animals	The study did not involve wild animals
Field-collected samples	The study did not involve samples collected from the field
Ethics oversight	All experiments were carried out in accordance with the accepted standards of humane animal care under the regulation of the Animal (Scientific Procedures) Act UK 1986 and were approved by the University of Edinburgh animal ethics committee.

Note that full information on the approval of the study protocol must also be provided in the manuscript.
