# Supplementary material for: Adaptive prospective optical gating enables day-long 3D time-lapse imaging of the beating embryonic zebrafish heart
Source: Nat Commun. 2019 Nov 15;10:5173. doi: 10.1038/s41467-019-13112-6 (PMC6858381; doi:10.1038/s41467-019-13112-6)
Supplement: Supplementary file 2 — Peer Review File [file 41467_2019_13112_MOESM2_ESM.pdf]

Editorial Note: This manuscript has been previously reviewed at another journal that is not operating a transparent peer review scheme. The manuscript was considered suitable for publication without further review at Nature Communications.
